# Paths to hippocampal damage in neuromyelitis optica spectrum disorders

**DOI:** 10.1111/nan.12893

**Published:** 2023-03-06

**Authors:** Mona Zakani, Magdalini Nigritinou, Markus Ponleitner, Yoshiki Takai, Daniel Hofmann, Sophie Hillebrand, Romana Höftberger, Jan Bauer, Balint Lasztoczi, Tatsuro Misu, Gregor Kasprian, Paulus Rommer, Monika Bradl

**Affiliations:** ^1^ Division of Neuroimmunology, Center for Brain Research Medical University of Vienna Vienna Austria; ^2^ Department of Neurology Medical University of Vienna Vienna Austria; ^3^ Department of Neurology Tohoku University Graduate School of Medicine Sendai Japan; ^4^ Department of Neurology, Division of Neuropathology and Neurochemistry Medical University of Vienna Vienna Austria; ^5^ Division of Cognitive Neurobiology, Center for Brain Research Medical University of Vienna Vienna Austria; ^6^ Division of Biomedical Imaging and Image‐guided Therapy Medical University of Vienna Vienna Austria

**Keywords:** aquaporin 4, hippocampus, microglial activation, neuromyelitis optica, retrograde degeneration

## Abstract

**Aims:**

Many patients with neuromyelitis optica spectrum disorders (NMOSD) suffer from cognitive impairment affecting memory, processing speed and attention and suffer from depressive symptoms. Because some of these manifestations could trace back to the hippocampus, several magnetic resonance imaging (MRI) studies have been performed in the past, with a number of groups describing volume loss of the hippocampus in NMOSD patients, whereas others did not observe such changes. Here, we addressed these discrepancies.

**Methods:**

We performed pathological and MRI studies on the hippocampi of NMOSD patients, combined with detailed immunohistochemical analysis of hippocampi from experimental models of NMOSD.

**Results:**

We identified different pathological scenarios for hippocampal damage in NMOSD and its experimental models. In the first case, the hippocampus was compromised by the initiation of astrocyte injury in this brain region and subsequent local effects of microglial activation and neuronal damage. In the second case, loss of hippocampal volume was seen by MRI in patients with large tissue‐destructive lesions in the optic nerves or the spinal cord, and the pathological work‐up of tissue derived from a patient with such lesions revealed subsequent retrograde neuronal degeneration affecting different axonal tracts and neuronal networks. It remains to be seen whether remote lesions and associated retrograde neuronal degeneration on their own are sufficient to cause extensive volume loss of the hippocampus, or whether they act in concert with small astrocyte‐destructive, microglia‐activating lesions in the hippocampus that escape detection by MRI, either due to their small size or due to the chosen time window for examination.

**Conclusions:**

Different pathological scenarios can culminate in hippocampal volume loss in NMOSD patients.

## Graphical abstract

The hippocampus may become compromised by astrocyte injury and local effects of microglial activation and neuronal damage. The hippocampus may also become compromised when large tissue‐destructive lesions in optic nerves or spinal cord initiate retrograde neuronal degeneration affecting different axonal tracts and neuronal networks. It remains unclear whether remote lesions and associated retrograde neuronal degeneration on their own are sufficient to cause extensive hippocampal volume loss, or whether they act in concert with small astrocyte‐destructive lesions in the hippocampus.

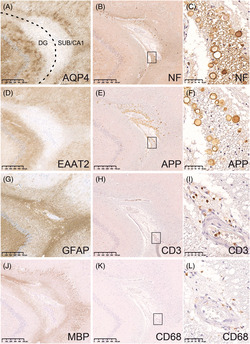

**Highlights**

- We identified different mechanisms contributing to hippocampal damage in NMOSD and its experimental models.
- The hippocampus may become compromised by the initiation of astrocyte injury and the subsequent local effects of microglial activation and neuronal damage.
- Loss of hippocampal volume was also observed in patients with large tissue‐destructive lesions in the optic nerves or spinal cord. Such lesions may initiate retrograde neuronal degeneration affecting different axonal tracts and neuronal networks.
- It remains to be seen whether remote lesions and associated retrograde neuronal degeneration on their own are sufficient to cause extensive volume loss in the hippocampus, or whether they act in concert with small astrocyte‐destructive, microglia‐activating lesions in the hippocampus that escape detection by MRI, either due to their small size or due to the chosen time window for examination.

## INTRODUCTION

Neuromyelitis optica spectrum disorders (NMOSD) are severe inflammatory diseases of the central nervous system (CNS) most frequently associated with the presence of pathogenic serum autoantibodies against the water channel aquaporin 4 (AQP4). In the course of these disorders, AQP4‐specific antibodies (AQP4‐abs) gain entry to the CNS, bind to astrocytes and initiate the destruction of these cells by complement‐dependent and/or antibody‐dependent cellular cytotoxicity, predominantly in the optic nerves and spinal cord. Astrocyte destruction is then rapidly followed by irreparable damage to axons. This process is responsible for the most feared consequence of an onset attack or relapses in NMOSD, that is, permanent disability. Over the last years, however, other possible consequences of NMOSD lesions came into the focus of attention: cognitive impairment, which is seen in 50%–75% of NMOSD patients and involves memory, processing speed, attention and depressive symptoms [1, 2, 3, 4, 5, 6, 7, 8, 9, 10, 11, 12, 13, 14, 15]. It was shown that cognitively impaired NMOSD patients and those with verbal and visual memory impairment had hippocampal atrophy [16] and that many of the memory deficits described in NMOSD patients affect hippocampus‐dependent short‐term and immediate memory [4, 6], whereas NMOSD patients with attentive or executive impairment had preserved hippocampal volumes [16]. Moreover, a large study of NMOSD patients involving extensive neuropsychological testing and magnetic resonance imaging (MRI) described hippocampal volume loss as the main predictor for cognition [8]. Essentially, these findings were confirmed by some research groups [16, 17, 18, 19], whereas others could not find any relation between cognition and hippocampal volume [13, 20]. These discrepant findings need to be explained, but to date, not a single study has shown whether, how and to what extent the systemic presence of AQP4‐specific antibodies induce damage to the hippocampus. Here, we addressed these discrepancies and performed pathological and MRI studies on the hippocampi of NMOSD patients, combined with detailed immunohistochemical analysis of hippocampi from experimental models of NMOSD.

## MATERIALS AND METHODS

### Patients

For pathological studies, hippocampi of four NMOSD patients were available; two of them (NMO01 and NMO02; Table 1) showed pathological changes. MRI studies were performed on 10 female NMOSD patients (NMO03–NMO17; mean age 45.4 ± 13.1 years; see Table 1 for detailed clinical information) and on 9 age‐matched healthy female controls (mean age 39.3 ± 13.0 years); age difference to NMOSD patients was statistically not significant (*p* = 0.33, two‐sample *t*‐test).

**TABLE 1:** Clinical information about the NMOSD patients included in this study.

ID	Disease duration (years)	Disease history (DH), therapeutic history (TH) and AQP4‐abs status (abs)	Disease stage at the time of death or of MRI	Pathological study (P) or MRI (M); location of lesions at this time point
NMO01	21	(DH) Bilateral optic neuritis, followed by several episodes of neurological symptoms including area postrema syndrome, optic neuritis and myelitis. Patient developed a hallucination delusion state and dementia (mild cognitive impairment) progressing to delirium. Brain and hippocampus CT was normal without marked atrophy, unlike Alzheimer's disease. Her mini‐mental state examination (MMSE) was at 27 points and gradually worsened; recent memory disturbance was not marked. (TH) In the last years of life Risperidone and Seroquel. (abs) AQP4‐abs positive.	Found dead at home. Cause of death unknown.	(P) Typical pathology of NMOSD with lesions defined by AQP4 loss and GFAP loss in the spinal cord. MRI not available.
NMO02	0.2	(DH) Paraesthesia of left arm and paresis of both arms; rapid development and deterioration of progressive tetraparesis; also CREST syndrome with Raynaud symptoms, Sjögren's syndrome and Hashimoto thyroiditis. (TH) Cortisone. (abs) AQP4‐abs positive.	First attack; death from cardiorespiratory failure.	(P) Single lesions with loss of AQP4 and loss of GFAP reactivity in the thalamus lateral to the substantia nigra, in crus cerebri, the tegmentum pontis, and the floor of the 4th ventricle, and the medulla oblongata at the site of the lemniscus medialis. Multiple, in part necrotic lesions with loss of AQP4 and loss of GFAP reactivity were found throughout the entire spinal cord, mostly affecting white matter.
NMO03	15	(DH) Optic neuritis, followed 1 year later by a sensorimotor relapse. Last relapse: Visual loss + sensory loss of the left upper extremity. (TH) 3 monthly cyclophosphamide infusions. (abs) AQP4‐abs positive.	Remission; complete visual loss left, partial visual loss right, left hemiparesis; EDSS: 3.5.	(M) 5 years after last relapse; atrophy of optical nerves and chiasma (no acute component), LETM C2–C4 (scarred‐gliotic post relapse), white matter lesions: ‐ right subcentrally, no contrast enhancement ‐ right postcentrally/opercular, positive contrast enhancement
NMO04	3	(DH) Lesions only in the spinal cord. (TH) Tocilizumab, multiple plasma exchanges. (abs) AQP4‐abs positive.	Relapse; no involvement of cranial nerves, paraparesis (requires assistance for walking), hypoesthesia below Th10; EDSS: 6.5.	(M) Lesions only in the spinal cord, no cerebral lesions.
NMO05	13	(DH) NMO. (TH) Rituximab, tocilizumab. (abs) AQP4‐abs positive.	Remission; spastic paraparesis since motor relapse, no visual symptoms; EDSS 6.5.	(M) Clinically stable at time of MRI; lesions only in the spinal cord (condition after LETM C2–C5), no cerebral lesions.
NMO06	1	(DH) NMO after first relapse with sensory impairment below Th5; relapse and progression with additional spinal lesions. (TH) Rituximab, tocilizumab. (abs) AQP4‐abs positive.	Remission; EDSS 0.0.	(M) Multiple spinal lesions, no cerebral lesions.
NMO07	1?	(DH) AQP4‐Ab‐positive NMOSD and AChR‐Ab‐positive myasthenia gravis; no clear clinical onset with visual acuity of 0.05 of the right eye with severe atrophy of the optic nerve, additionally, oculomotor symptoms of no definite origin, most probably due to the myasthenia gravis. (TH) Not available. (abs) AQP4‐abs positive.	Remission; marked visual loss of the right eye; EDSS 3.0.	(M) Severe atrophy of the optic nerve, no cerebral demyelinating lesions.
NMO09	0	(DH) Large demyelinating lesion in the chiasma opticum with involvement of the optic nerve and optical tract. Isolated visual symptoms. (TH) Rituximab. (abs) AQP4‐abs positive.	Relapse; acute relapse with visual loss and bitemporal hemianopia; EDSS 3.0.	(M) Chiasmic lesion, only small cerebral white matter lesions (<3 mm, primarily microangiopathic), no spinal lesions.
NMO11	13	(DH) First manifestation with a right‐side hemiparesis, followed several years later by visual loss of the right eye and with loss of ability to stand; autoimmune thyroiditis. (TH) Mycophenolatmofetil. (abs) AQP4‐abs titre at time of MRI unknown, now AQP4‐abs negative.	Relapse; partial visual loss of the left eye (new), complete visual loss of the right eye, gait ataxia and paresis of the right leg; EDSS 4.0.	(M) Severe atrophy of the chiasma opticum and moderate atrophy of both optic nerves, singular small (<3 mm) cerebral white matter lesions (primarily microangiopathic).
NMO13	1	(DH) Area postrema syndrome only. (TH) Rituximab. (abs) AQP4‐abs positive.	(prolonged) Relapse; area postrema syndrome; EDSS 0.0.	(M) Area postrema lesion (FLAIR hyperintense), no other abnormalities.
NMO16	11	(DH) Relapses with bilateral optic neuritis; additionally, condition after thyroiditis. (TH) Rituximab, tocilizumab; untreated at time of relapse. (abs) AQP4‐abs positive.	Relapse; complete visual loss of the left eye. No other neurologic symptoms; EDSS 3.0.	(M) Left optic nerve lesion with contrast enhancement, mild atrophy of optic tracts, increasing periventricular lesions on both sides, juxtacortical lesion in the superior right parietal lobe, small white matter lesions, singular, small microbleeds.
NMO17	4	(DH) Initially diagnosed as highly active RRMS, disease progression and final diagnosis as NMOSD. (TH) Natalizumab (!), then rituximab. (abs) AQP4‐abs positive.	Remission; tetraparesis and marked loss of vision; EDSS 6.5.	(M) Large periventricular lesions (already black holes in T1), most prominently left frontally, lesion in the splenium of the corpus callosum, lesion in the left chiasma opticum, atrophy of both optic nerves, multiple spinal lesions, no contrast enhancement.

*Note*: All patients were female. Tissue samples of patients NMO01 and NMO02 were used for pathological studies, and patients NMO03–NMO17 were studied by MRI.

Abbreviations: AQP4, aquaporin 4; CT, computed tomography; EDSS, Expanded Disability Status Scale; GFAP, glial fibrillary acidic protein; LETM, longitudinally extensive transverse myelitis; MRI, magnetic resonance imaging; NMO, neuromyelitis optica; NMOSD, neuromyelitis optica spectrum disorders; RRMS, relapsing‐remitting multiple sclerosis.

### Animals

Seven‐ to 8‐week‐old Rowett Nude (RNU), Lewis (LEW) and Brown Norway (BN) rats were obtained from Charles River Wiga (Sulzfeld, Germany) and housed under standardised conditions in the Decentral Facilities of the Institute for Biomedical Research (Medical University Vienna).

### The ‘antibody‐only’ experimental model

The monoclonal murine AQP4‐specific antibody E5415A [21, 22, 23], termed ‘AQP4‐abs’ throughout the manuscript, and control mouse IgG (Sigma, Vienna, Austria) were used in concentrations of 1 mg/mL in phosphate‐buffered saline (PBS). The antibodies were injected intraperitoneally for 5 consecutive days, and the animals were scored daily for the presence of clinical symptoms. Twenty‐four hours after the last injection, the rats were killed with CO_2_. Subsequently, serum was collected for the determination of antibody titres, and transcardial perfusion was undertaken with 4% paraformaldehyde (PFA) in PBS. Optic nerves, brains and spinal cords were dissected, postfixed in 4% PFA for 24 h and embedded in paraffin for histological analysis. Please note that we re‐evaluated the pathological samples of AQP4‐abs‐injected LEW and RNU rats described before [24] and made additional injections into BN rats, using the same batch of the AQP4‐abs.

### The ‘antibody plus T cell’ experimental models

Here, only LEW rats were used, because this strain of rat is able to mount T cell responses against a wide range of CNS antigens (see below), in contrast to BN rats [25] and to RNU rats lacking thymus‐dependent T cells implied in the pathogenesis of CNS inflammation [26, 27].

Activated CNS antigen‐specific T cells recognising myelin basic protein (MBP) [28, 29, 30], myelin oligodendrocyte glycoprotein (MOG) [29], S100beta (S100β) [29], AQP4 (AQP4_268–285_ [31]) or glial fibrillary acidic protein (GFAP; amino acid sequence SRMTPPLPARVDFSL = GFAP_38–52_) were injected into LEW rats on Day 0. At the onset of clinical symptoms, the pathogenic monoclonal murine AQP4‐specific antibody E5415A [21, 22, 23], termed ‘AQP4‐abs’, or NMOSD patient‐derived IgG containing AQP4‐specific antibodies, termed ‘NMO‐IgG’, were injected intraperitoneally. AQP4‐abs were injected in concentrations between 0.5 and 1.0 mg in 1‐mL PBS, 1× per animal. NMO‐IgGs were injected at a concentration of 10 mg in 1‐mL PBS, 1× per animal. The animals were sacrificed by CO_2_ inhalation, 24 (most animals) or 48 h (only selected cases) after antibody transfer. Please note that we re‐evaluated the pathological samples of LEW rats described before [24, 28, 29, 30, 31] and included additional animals receiving GFAP_38–52_‐specific T cells.

### Immunohistochemistry

#### Animal samples

All immunohistochemical procedures on the rat tissues have been performed as described [24], after antigen retrieval by steaming deparaffinised and rehydrated tissue sections for 1 h in 1‐mM ethylenediaminetetraacetic acid (EDTA) in 10‐mM Tris buffer (pH 8.6) (E) or by incubating them for 15 min in 0.03% protease‐type XXIV, subsequently indicated by P, respectively. The following antibodies were used: as primary antibodies, polyclonal rabbit anti‐rat AQP4 (1:250; E; Sigma‐Aldrich, Vienna, Austria), rabbit anti‐rat C9neo (1:2000; P [32]), monoclonal rabbit anti‐human CD3 (cross‐reactive to rat CD3; 1:2000; E; Thermo Scientific, Vienna, Austria), polyclonal rabbit anti‐cow GFAP (cross‐reactive with rat; 1:3000; E; DakoCytomation, Glostrup, Denmark), monoclonal mouse anti‐rat ED1 (1:10,000; E; Thermo Scientific), donkey anti‐rat IgG (1:1500; P; Jackson ImmunoResearch, West Grove, PA, USA), rabbit anti‐calbindin D‐28k (1:2000; E; Swant, Marly, Switzerland), mouse anti‐MHC class II RT1B (clone OX6; 1:250; E; Serotec, Oxford, UK), rabbit anti‐ionised calcium‐binding adapter molecule 1 (Iba‐1; E; 1:10,000; Wako, Neuss, Germany) and mouse anti‐synaptophysin Sy38 (1:1000; E; DakoCytomation). For conventional microscopy, biotinylated donkey anti‐rabbit (1:1000–1:2000, Jackson ImmunoResearch), biotinylated sheep anti‐mouse (1:500, Jackson ImmunoResearch) and biotinylated donkey anti‐sheep/goat (1:200, Amersham GE Healthcare) were used as secondary antibodies, and the reactions were completed with the AEC system (for C9neo) or by exposure to the avidin–peroxidase complex (1:200, Amersham GE Healthcare) and subsequent visualisation with 3,3′‐diaminobenzidine tetrahydrochloride (DAB; Sigma‐Aldrich) containing 0.01% hydrogen peroxide (all other antibodies). Finally, the tissue sections were counterstained with haematoxylin and mounted in geltol (sections developed with the AEC system), or dehydrated and mounted in Eukitt© (Merck, Darmstadt, Germany) (all other sections).

Fluorescent triple labelling was performed for ED1, synaptophysin and Iba‐1. The ED1 antibody (1:5000) was applied overnight, followed by a corresponding biotinylated secondary system and tyramide enhancement. Slides were then steamed again in EDTA (0.05 M) in Tris buffer (0.01 M, pH 8.5) for 30 min, followed by 1‐h incubation with Cy2‐conjugated streptavidin (Jackson ImmunoResearch). In a second overnight incubation, anti‐synaptophysin Sy38 (DakoCytomation, 1:50) and Iba‐1 (Wako, 1:1000) primary antibodies were applied to the tissue together, followed by Cy3‐conjugated donkey anti‐mouse (1:100, Jackson ImmunoResearch) and Cy5‐conjugated donkey anti‐rabbit (1:100, Jackson ImmunoResearch) secondary antibodies. Fluorescent preparations were examined using a confocal laser scan microscope (Leica SP5, Leica, Mannheim, Germany) equipped with lasers for 504, 488, 543 and 633 nm of excitation. Scanning for Cy2 (488 nm), Cy3 (543 nm) and Cy5 (633 nm) was performed sequentially to rule out fluorescence bleed‐through.

#### Patient samples

For immunohistochemical analysis, paraffin‐embedded sections were deparaffinised in xylene, rehydrated in ethanol and rinsed with PBS.

For NMO01, antigen retrieval was performed by heating the sections at an adequate temperature for the prescribed length of time in the heat retrieval solution ‘Diva Decloaker’ (Biocare Medical, Concord, CA, USA) in a decloaking chamber (model DC2002, Biocare Medical). After blocking non‐specific binding with 10% goat serum for 15 min at room temperature, the slides were covered and incubated with primary antibodies at 4°C overnight. We used primary antibodies as follows: anti‐AQP4 antibody (Santa Cruz Biotechnology, Dallas, TX, USA), anti‐GFAP antibody (Sanbio Research Diagnostics, Uden, Netherlands), anti‐EAAT2 (Cell Signaling Technology, Danvers, MA, USA), anti‐CD68 (DakoCytomation/Agilent, Santa Clara, CA, USA), anti‐MBP (DakoCytomation/Agilent), anti‐MOG (Abcam, Cambridge, UK), anti‐NFP (Merck Millipore, Tokyo, Japan) and anti‐APP (Merck Millipore).

After the incubation with primary antibodies, the slides were washed with PBS and incubated with 30% methanol/PBS containing 1% H_2_O_2_ for 20 min to block endogenous peroxidase, after which they were washed three times with PBS. Secondary antibodies were applied and incubated for 40 min at room temperature. For staining, diaminobenzidine tetrahydrochloride (DAB) (brown) was used for the horseradish peroxidase (HRP) system and fuchsin (DacoCytomation, CA) (red) or Vector blue (Vector, CA) (blue) for the alkaline phosphatase (AP) system. Selected sections were counterstained with haematoxylin (blue) or nuclear fast red (Vector) (red). For double staining, HRP and AP were combined.

For NMO02, antigen retrieval was performed by heating the sections in a commercial household steamer for 1 h in 10‐mM citrate buffer pH 6.0 (anti‐APP) or 1‐mM EDTA in 10‐mM Tris pH 9.0 (anti‐CD68, DakoCytomation/Agilent; anti‐EAAT2, Abcam). For staining with anti‐AQP4 (Merck/Sigma), no pre‐treatment was necessary. After the pre‐treatment, the sections were washed in 0.1‐M PBS and incubated for 20 min at room temperature with 10% fetal calf serum (FCS) in 1× Dako Wash Buffer to reduce non‐specific background staining. Then, the sections were incubated overnight at 4°C with the primary antibodies diluted 1:1000 (anti‐APP), 1:250 (anti‐AQP4, anti‐EAAT2) or 1:100 (CD68) in 1× Dako Wash Buffer containing 10% FCS. Afterwards, the sections were washed 3× in Tris‐buffered saline (TBS) and incubated for 1 h at room temperature with biotinylated anti‐mouse (to detect the primary antibodies anti‐APP and CD68) or biotinylated anti‐rabbit antibodies (to detect anti‐AQP4 and anti‐EAAT2). This incubation was followed by three washing steps in TBS before the sections were exposed for 1 h at room temperature to peroxidase‐conjugated streptavidin (Jackson ImmunoResearch, Cambridge, UK) diluted 1:100 in 1× Dako Wash Buffer containing 10% FCS.

Sections of an age‐matched human brain were used as control.

### Quantifications and statistical analysis

Single coronal brain sections of individual animals were used in the range of −2.12 → −3.60 mm from Bregma for the dorsal hippocampus and in the range of −4.16 → −6.72 mm from Bregma for the ventral hippocampus, using the stereotactic coordinates of Paxinos and Watson as guidelines [33]. The sections were analysed with the viewing software NDP.View 2.8.24 for digital slide observation (free edition from Hamamatsu photonics k.k.), using the freehand tool for annotation of the areas of the dorsal and ventral hippocampi and the areas with AQP4 loss located within these sites. Statistical analyses were performed with the IBM SPSS Statistics (Version 27) package, and the graphs were plotted in GraphPad Prism (Version 6).

### MRI

#### Image acquisition

Standard diagnostic MRI scans in the NMOSD cohort were retrospectively included after visual quality assessment by a neuroradiologist (GK). For analysis in this study, only coronary T2‐weighted scans performed at 3.0 Tesla with a slice thickness of ≤3 mm were included. All healthy control subjects received MRI scans with identical protocols. For details, see Table S1.

#### Image and statistical analysis

After anonymisation and export in DICOM format, co‐registration and realignment of the coronary T2 images to the standard MNI152T1_1mm matrix (1 mm × 1 mm × 1 mm) was done with the ITK‐SNAP application (www.itksnap.org [34]). Volumetry of the left and right hippocampi was performed with FSL FIRST ([35]) and subsequent manual correction of each image by MP in ITK‐SNAP. Finally, the mean volume of the left and right hippocampi was used for statistical analysis, which was done in IBM SPSS v27.0 (SPSS Inc, Chicago, IL, USA). Normal distribution was assessed and confirmed using the Kolmogorov–Smirnov method. Volume means between the two groups were compared with a two‐sided, independent sample *t*‐test, considering a *p*‐value of <0.05 significant.

## RESULTS

### Lesions with AQP4 loss may form in the hippocampus of human NMOSD patients and rats with experimental NMOSD‐like disease

**FIGURE 1:**
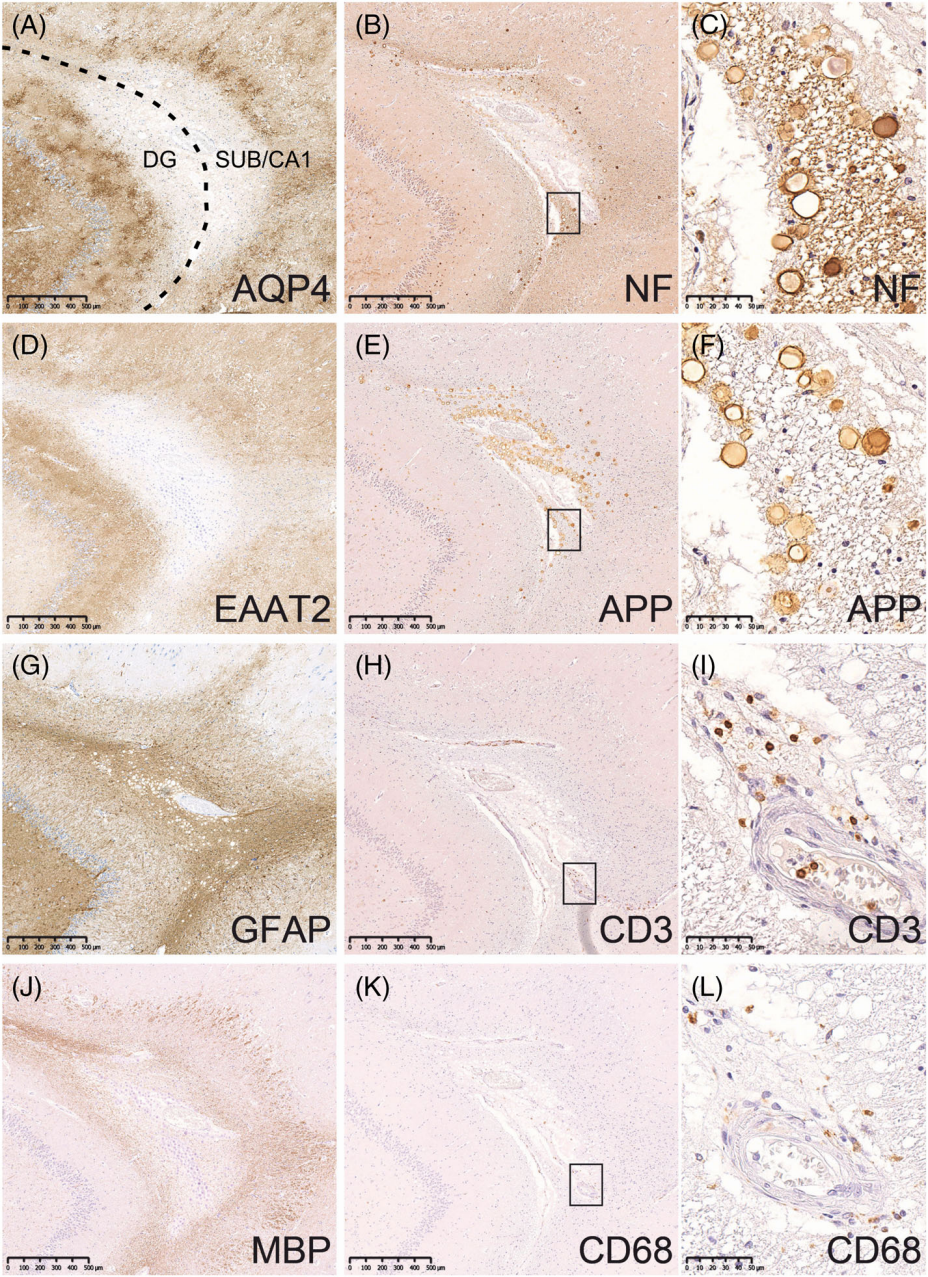
Hippocampal lesion with aquaporin 4 (AQP4) loss in human neuromyelitis optica spectrum disorders. Patient NMO01. Consecutive sections of the medial temporal lobe at the level of the hippocampus were stained with antibodies specific for AQP4 (A), neurofilament (NF) (B, boxed area enlarged in C), EAAT2 (D), amyloid‐beta precursor protein (APP) (E, boxed area enlarged in F), glial fibrillary acidic protein (GFAP) (G), CD3 (H, boxed area enlarged in I), myelin basic protein (MBP) (J) and CD68 (K, boxed area enlarged in L). All positive reaction products are brown. Please note that the corpora amylacea show non‐specific reactivity for NF and APP. The tissue was counterstained with haematoxylin to show nuclei (blue). The dashed line indicates the meninges; bar = 500 μm (A, B, D, E, G, H, J, K) and 50 μm (C, F, I, L). CA1, Cornu Ammonis 1; DG, dentate gyrus; SUB, subiculum.

The hippocampus of patient NMO01 contained a small subpial lesion, about 1.0 mm wide and 1.5 mm long, with loss of AQP4 and loss of EAAT2 reactivity (Figure 1). This lesion located at the border between the subiculum/Cornu Ammonis (CA)1 and dentate gyrus (DG) and was demyelinated, as evidenced by the absence of MBP (Figure 1) and MOG reactivity (data not shown). Neurofilament (NF) positive axons were lost from the lesion core, and many corpora amylacea (showing non‐specific reactivity in the NF and amyloid‐beta precursor protein [APP] staining) were present in the periphery of the lesion (Figure 1). The low numbers of CD3^+^ T cells and CD68^+^ macrophages, the absence of CD68^+^ microglia in the periphery of the lesion, the lack of neutrophils (Figure S1), the absence of antibody and/or complement depositions and the presence of GFAP^+^ astrocytes jointly indicated an older, chronic lesion stage with evidence of astrogliosis (Figure 1). Such lesions are classified as pattern D NMOSD lesions [36, 37]. Typical NMOSD lesions seem to be rare because we could not detect more of them in additional 11 hippocampi from NMOSD patients studied by pathological analysis (*n* = 1) or MRI (*n* = 10) (data not shown).

**FIGURE 2:**
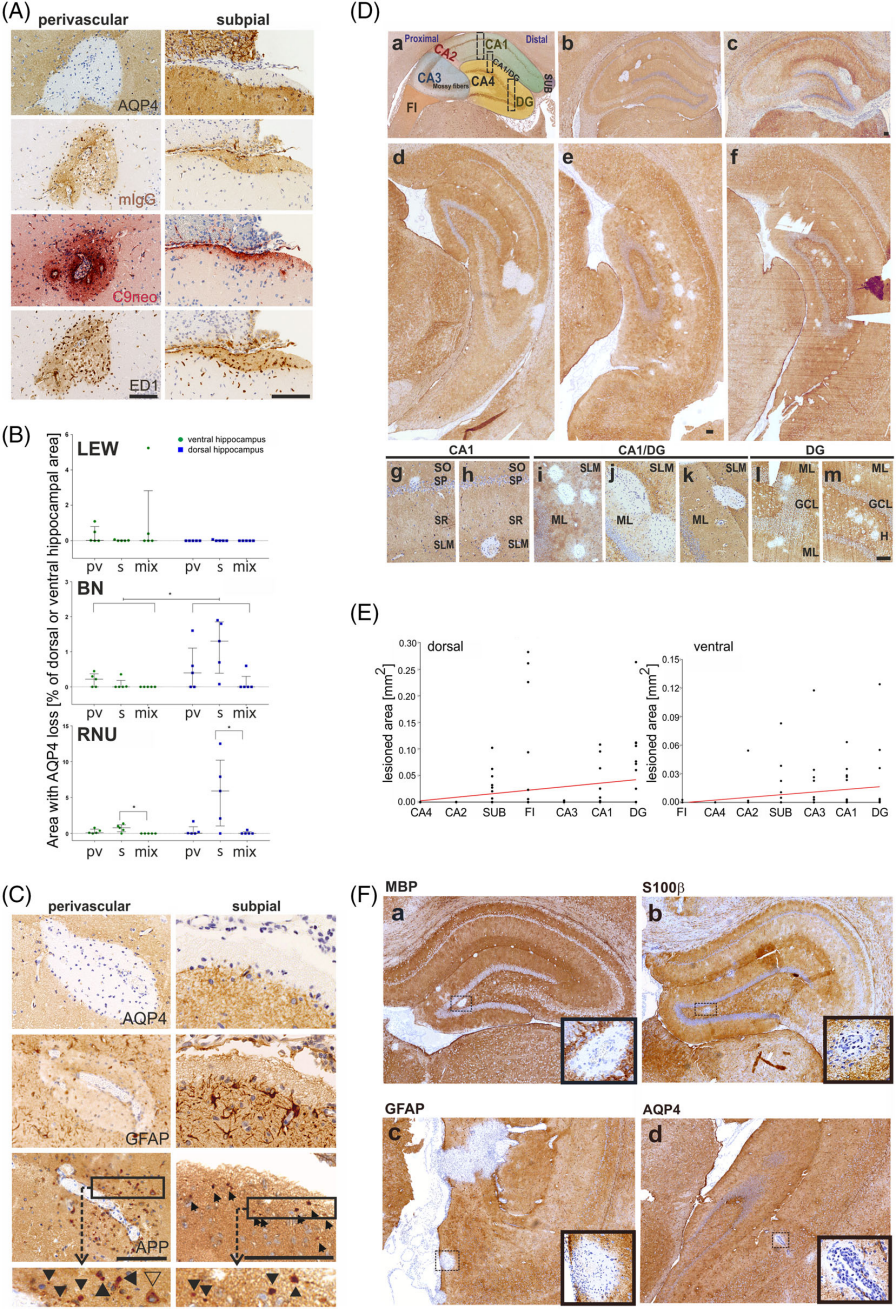
Hippocampal lesions with aquaporin 4 (AQP4) loss in experimental neuromyelitis optica spectrum disorders. (A) Cellular and molecular substrates for lesion formation. Consecutive coronal brain sections at the level of the hippocampus were stained with antibodies specific for AQP4, murine IgG (mIgG) to reveal bound pathogenic antibodies on the surface of astrocytes, complement C9neo (C9neo) to reveal the terminal membrane attack complex as an indicator for complement‐dependent (cellular) cytotoxicity and the antibody ED1 specific for activated microglia/macrophages needed for antibody‐dependent cellular cytotoxicity. With exception of complement C9neo, where a positive reaction product is shown in red, all other antibody reaction products are shown in brown. Counterstaining was done with haematoxylin to reveal nuclei (blue). Perivascular and subpial lesions are shown. All lesions shown here are derived from Brown Norway (BN) rats, analysed 24 h after daily intraperitoneal injections of AQP4‐abs for 5 consecutive days, and are representative of all other rat strains treated the same way. Bars = 100 μm. (B) Statistical evaluation of the within‐rat‐group differences in the distribution of hippocampal lesion phenotypes, along the dorsal–ventral hippocampal axis. For normalisation, areas with AQP4 loss are expressed as a percentage of the total dorsal (blue data points) or ventral hippocampal area (green data points) containing these lesions. Each data point represents the lesioned area value for one rat (*n* = 5 per rat strain). The plots show the median of AQP4 loss (horizontal lines) with an interquartile range (whiskers). To evaluate the within‐rat‐group differences in phenotypes of hippocampal lesions, the data were analysed with the related‐samples Friedman's two‐way analysis of variance by ranks test. For the within‐rat‐group differences of the total lesion area between the ventral and dorsal hippocampi, the related‐samples Wilcoxon signed rank test was used. These non‐parametric statistical tests accounted for the small sample size per group, the non‐normally distributed data and the presence of outliers in the data sets. Due to the small sample size, the exact significance values were taken into account and the data were further analysed with Dunn's post hoc test followed by Bonferroni's corrections for multiple testing. Pairwise comparisons were calculated only if the omnibus test returned significant results. Statistically significant results after pairwise comparisons are depicted with asterisks (* = 0.01 ≤ *p* ≤ 0.05). pv, perivascular lesions; s, subpial/subependymal lesions; mix, lesions with subpial or subependymal and perivascular features. (C) Consequences of AQP4 loss. Consecutive coronal brain sections at the level of the hippocampus were stained with antibodies specific for AQP4, glial fibrillary acidic protein (GFAP) and amyloid‐beta precursor protein (APP). In both perivascular and subpial hippocampal lesions, astrocytes are lost as evidenced by loss of AQP4 and GFAP reactivity, and APP^+^ axonal spheroids/endbulbs (black arrowheads) as markers for transient and permanent neuronal injury are visible. Boxes indicate areas of the tissue shown in higher magnification below, where black arrowheads point to APP^+^ axonal spheroids/endbulbs and the open arrowhead marks an APP^+^ neuronal cell body. With exception of complement C9neo, where a positive reaction product is shown in red, all other antibody reaction products are shown in brown. Counterstaining was done with haematoxylin to reveal nuclei (blue). All lesions shown here are derived from BN rats, analysed 24 h after daily intraperitoneal injections of AQP4‐abs for 5 consecutive days, and are representative of all rat strains treated the same way. Bars = 100 μm. (D) Hippocampal subfields containing lesions with AQP4 loss. Coronal brain sections at the level of the hippocampus were stained with antibodies specific for calbindin (a) and AQP4 (brown reaction product, b–m) and counterstained with haematoxylin to reveal the nuclei (blue). (a) derived from untreated Lewis (LEW) rats, whereas (b, c, e, f, i, j, l, m) from Rowett Nude (RNU) rats, (d) from an LEW rat and (g, h, k) from BN rats. Apart from the animal shown in (a), all other animals were analysed 24 h after daily intraperitoneal injections of AQP4‐abs for 5 consecutive days. In (a), the following subfields of the dorsal hippocampus of an untreated LEW rat are outlined: Fimbria (FI), Cornu Ammonis (CA) areas CA1–CA4, dentate gyrus (DG) and subiculum (SUB). The boxed areas define regions shown in more detail in (g–m). In (b–f), coronal sections of the dorsal (b, c) and ventral (d–f) hippocampi show lesions with AQP4 loss. In (g–m), lesions with AQP4 loss are shown in higher magnifications. The following hippocampal layers are indicated: Stratum oriens (SO), stratum pyramidale (SP), stratum radiatum (SR), stratum lacunosum moleculare (SLM), molecular layer (ML), granule cell layer (GCL) and hilus (H). All bars: 100 μm. (E) Correlation analyses between hippocampal subfield size and lesioned area size are shown for both the dorsal and ventral hippocampi. Data from all rat strains were pooled for these analyses. On the x‐axis (independent variable), the different hippocampal subfields were assigned and depicted in an ordinal and numerical scale ranging from 1 to 7, in ascending size order. After correlation analysis was done, these numbers were translated back to the subfield name for easier representation in the graph. On the y‐axis (dependent variable), the values of the lesioned area (= area with AQP4 loss) were given in mm^2^. The relationship between the two correlated factors seemed to approximate a line (they did not show a curved or parabolic shape). Therefore, a linear correlation statistical approach was used. Because the independent variable was measured in ordinal scale and because the data were not normally distributed, the non‐parametric Spearman rank order correlation coefficient was used, which showed a ‘moderate to low’ yet statistically significant correlation between the two variables for both ventral (*r*_*s*_ [103] = 0.382, *p* < 0.0001, ****) and dorsal hippocampi (*r*_*s*_ [103] = 0.340, *p* = 0.0004, ***). (F) Central nervous system (CNS) antigen‐specific T cells pave the way for antibody entry to the hippocampus. Consecutive coronal brain sections at the level of the hippocampus were stained with antibodies against AQP4 (brown) and counterstained with haematoxylin to reveal nuclei (blue) to reveal lesions with loss of AQP4 reactivity. The tissue sections derived from LEW rats injected with T cells specific for myelin basic protein (MBP, a), S100beta (S100β) (b), glial fibrillary acidic protein peptide GFAP_38–52_ (c) or aquaporin 4 peptide AQP4_268–285_ (d) to induce CNS inflammation, followed by NMO‐IgG when the animals showed first clinical symptoms. The lesions were very small and are shown in higher magnification in the figure inlays. Bar = 100 μm.

We next resorted to the hippocampi of experimental rats to decipher the pathological mechanisms underlying the formation of lesions at this site. We first studied the ‘antibody‐only’ rat model of NMOSD with NMOSD‐typical lesions in the spinal cords and brains ([24] and Figure S2). 4/5 LEW, 4/5 RNU and 5/5 BN rats also displayed lesions with AQP4 loss in their dorsal and/or ventral hippocampi. These lesions had subpial/subependymal or perivascular locations, or representative mixtures thereof (Figure 2), with subtle differences in size and location between the three different strains of rats. RNU rats had the largest areas with AQP4 loss and dominance of subpial/subependymal lesions (Figure 2). As a trend, this was also seen in the dorsal hippocampus of BN rats (Figure 2), whereas LEW rats almost exclusively displayed small areas with AQP4 loss in the ventral hippocampus (Figure 2).

The largest subfields in both dorsal and ventral hippocampi, that is, subiculum, DG, CA subfield CA1 and the CA1/DG border (fissure), contained the largest areas of AQP4 loss (Figures 2 and S3). Predominantly affected layers were the CA1 stratum lacunosum moleculare (SLM) and the DG molecular layer (ML), which contained lesions in 2/5 LEW rats, 4/5 BN rats and 4/5 RNU rats (Figure S3). Lesions with AQP4 loss were also found in the fimbria of the dorsal and the CA3 region of the ventral hippocampus, reflecting the different sizes of these subfields along the dorsal–ventral neuroaxis. Lesions in the smallest subfields, that is, in CA2 and CA4, were essentially absent (Figures 2 and S3).

Because the antibodies had been applied over several days, hippocampal lesions of different ages were present. Most hippocampal lesions were early‐active; that is, they were characterised by the presence of ED1^+^ macrophages/activated microglia (Figure 2) and neutrophils (Figure S4) and by the deposition of immunoglobulins suggesting the action of antibody‐dependent cellular cytotoxicity (ADCC). Some hippocampal lesions were older, essentially devoid of neutrophils, but still show profound AQP4 loss (Figure S4). Perivascular lesions showed profound complement C9neo deposition throughout the lesions, indicative of an additional, strong contribution of complement‐dependent cytotoxicity (CDC) to lesion formation, whereas subpial lesions displayed C9neo deposits well behind the lesion border, indicating a dominance of ADCC over CDC at these sites (Figure 2). Both perivascular and subpial/subependymal hippocampal lesions with AQP4 loss culminated in the loss of GFAP reactivity indicative of astrocyte loss and the presence of APP^+^ axon spheroids/endbulbs and APP^+^ neuronal cell bodies indicating neuroaxonal dysfunction/damage (Figure 2).

We next studied the hippocampi of the ‘antibody plus T cell’ experimental rat models of NMOSD and also found subpial/subependymal and deep perivascular lesions with AQP4 loss, in line with T cell and antibody passage across the blood–brain barrier and tissue entry from subarachnoid and perivascular spaces [38, 39]. Depending on the CNS antigen specificities of the T cells used to open the blood–brain barrier for the induction of CNS inflammation and AQP4‐abs entry into the CNS, hippocampal lesions formed in the range of 0% (MOG‐specific T cells) to 86% (S100β‐specific T cells) (Table 2). These lesions showed AQP4/GFAP loss (Figure 2) and contained IgG and complement deposits along with ED1^+^ activated microglia/macrophages. Early and late active lesions were seen, as evidenced by the presence or absence of neutrophils, respectively (Figure S4). Although these lesions could be found anywhere within the hippocampus, they were most frequently located in the CA1, CA1/DG, DG and CA3 subfields (Table 2 and Figure 2).

**TABLE 2:** Type and subfield location of lesions with AQP4 loss formed in Lewis rats after systemic injection of activated CNS antigen‐specific T cells and the murine monoclonal antibody E5415A (AQP4‐abs) or immunoglobulin preparations from five different AQP4‐antibody‐positive NMOSD patients (NMO‐IgGs 1–5).

Antigen specificity of T cells	Antibodies used	Analysed after antibody transfer (h)	# of rats with lesions/# of rats treated	Type of lesions (# of rats)	Subfields with lesions	For each T cell antigen: Rats with hippocampal lesions among all rats (%)
MBP	NMO‐IgG 1	24	1/5	pv	CA1, DG	
	AQP4‐abs	24	1/1	pv	CA1, CA2, CA3	
		30–40	1/1	pv, sp	CA1, CA2, CA3	
		48	4/5	pv (4), sp (3)	**CA1/DG, DG**, CA2, CA3, **SUB**	
	NMO‐IgG 2	48	1/3	pv	CA1	53.3%
MOG	NMO‐IgG 1	24	0/5	n.d.	n.d.	0.0%
AQP4_268–285_	NMO‐IgG 3	24	0/5	n.d.	n.d.	
		48	0/2	n.d.	n.d.	
		48	1/3	pv	CA1, CA2	
	AQP4‐abs	24	0/2	n.d.	n.d.	
	NMO‐IgG 4	24	0/5	n.d.	n.d.	
		48	1/5	pv	FI	
	NMO‐IgG 5	24	2/5	pv (2)	CA1, DG	14.8%
GFAP_38–52_	NMO‐IgG 4	24	2/11	pv (2), sp (2)	CA1, **DG,** CA3, SUB,	
	AQP4‐abs	24	2/3	pv (2), sp (2)	CA1, **DG,** CA1/DG, CA2, CA3, SUB, FI	28.6%
S100β	NMO‐IgG 1	24	5/5	pv (5), sp (3)	**CA1, DG, CA1/DG, CA2, CA3,** CA4	
	NMO‐IgG 4	24	1/2	pv	DG	85.7%

*Note*: In these models, NMO‐IgG or AQP4‐abs were injected 1×, and the animals were sacrificed after 24 or 48 h. One animal was found dead, after 30–40 h. Lesions with AQP4 loss formed perivascular (pv) and subpial (sp), and the numbers of animals per group forming such lesions are shown in brackets. Hippocampal subfields with lesions were fimbria (FI), Cornu Ammonis (CA) areas CA1–CA4, dentate gyrus (DG), the contact region between the CA1 area and the DG (CA1/DG) and subiculum (SUB). Subfields encountered more than once per group are shown in boldface.

Abbreviations: #, number of animals; AQP4, aquaporin 4; CNS, central nervous system; GFAP, glial fibrillary acidic protein; MBP, myelin basic protein; MOG, myelin oligodendrocyte glycoprotein; n.d., not done; NMO, neuromyelitis optica; NMOSD, neuromyelitis optica spectrum disorders; S100β, S100beta.

Cumulatively, these data suggested that

1. lesions with AQP4 loss can essentially form anywhere in the hippocampus,
2. location and size of such lesions correlate with the size of the hippocampal subfields and
3. hippocampal lesions with AQP4 loss form by the same cellular and molecular substrates (i.e., ADCC and CDC) as their counterparts found elsewhere in the CNS [24, 40].

### In the presence of AQP4‐abs, beginning and established hippocampal subependymal inflammation coincides with the appearance of dispersed activated microglia in the hippocampus

**FIGURE 3:**
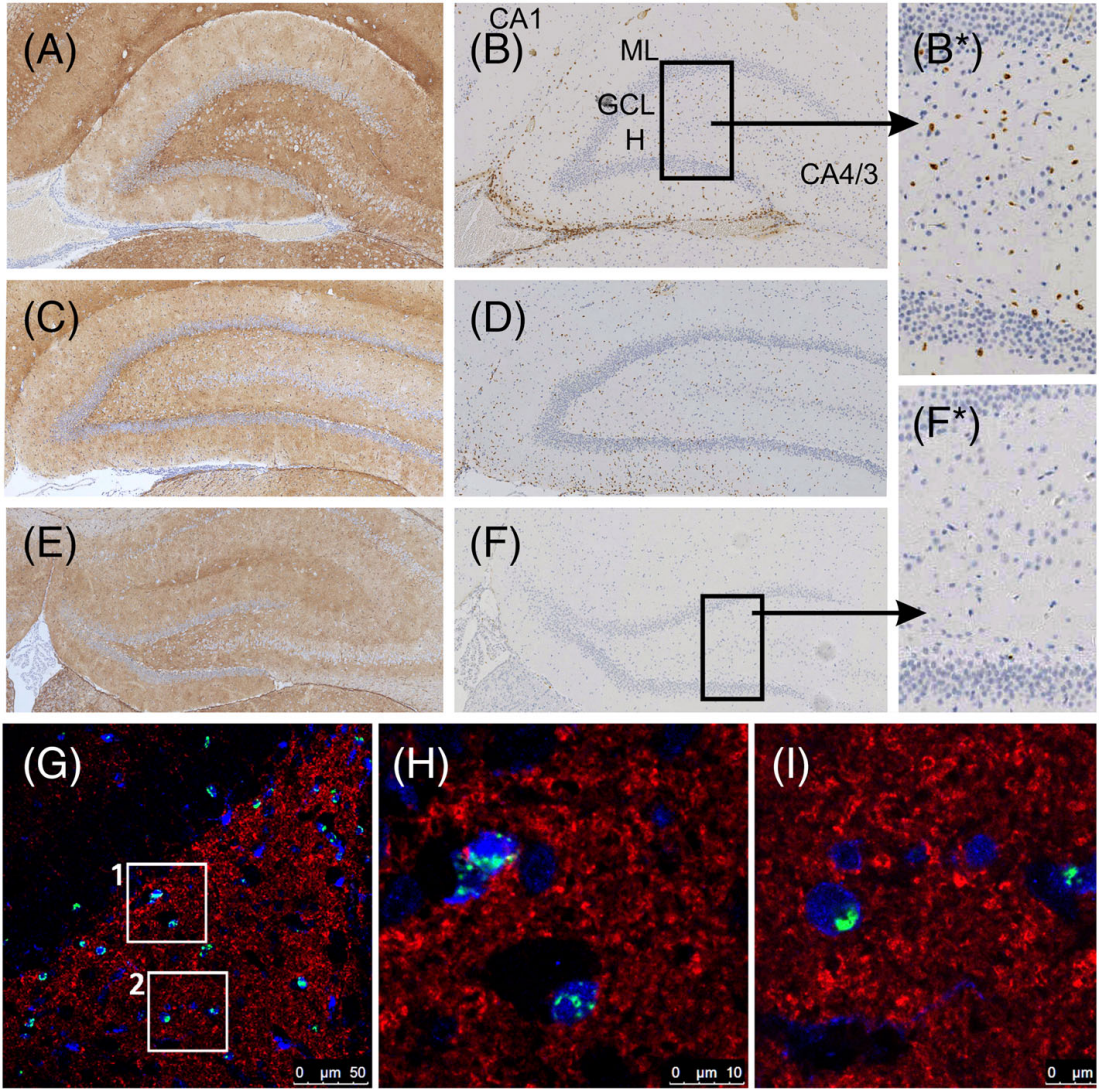
Hippocampal subependymal inflammation in AQP4‐abs^+^ rats coincides with the presence of dispersed activated microglia/macrophages in the dentate gyrus. Shown here are coronal sections of rats at the level of the hippocampus. All animals were analysed 24 h after daily intraperitoneal injections of AQP4‐abs for 5 consecutive days. (A) Rowett Nude rats. The sections were stained with aquaporin 4 (AQP4)‐specific antibodies (positive reaction product brown) to show the presence (A, C) or the absence (E) of remote subependymal lesions with AQP4 loss, and with the ED1 antibody to identify activated microglia/macrophages (B, D, F). Sections were counterstained with haematoxylin to show nuclei (blue). In the presence of remote subependymal lesions with AQP4 loss (A, C), single activated microglial cells are seen in the dentate gyrus (B, boxed area enlarged in B*; D). In the absence of subependymal lesions with AQP4 loss (F), no ED1‐positive activated microglia/macrophages could be observed (F, boxed area enlarged in F*). CA, Cornu Ammonis area; GCL, granule cell layer; H, hilus; ML, molecular layer. (G) Staining for ED1 (green), synaptophysin (red) and Iba‐1 (blue) in the hippocampus shows a paraventricular area with ED1^+^/Iba‐1^+^ (phagocytic) macrophages/activated microglia as well as ED1^−^/Iba‐1^+^ microglia. ED1^+^ cells in rectangles 1 and 2 are shown in higher magnification, respectively, in (H) and (I). Despite the phagocytic phenotype of the macrophages/activated microglia, no uptake of synaptophysin can be seen.

When we were studying ongoing and established subependymal lesions with AQP4 loss in the hippocampus of the ‘antibody‐only’ rats, we noticed that affected animals displayed areas with scattered activated microglia/macrophages far beyond the lesion border, mostly in the DG and subiculum (Table 2). The activated microglial cells/macrophages had an amoeboid phenotype, were ED1^+^ (Figures 3 and S5) and did not express MHC class II molecules (data not shown). There was no evidence for the binding of AQP4‐abs to astrocytes and no evidence of complement deposition at the sites of the scattered activated microglial cells/macrophages (Figure 3). We also did not find apoptotic nuclei or APP^+^ axonal spheroids/endbulbs in these areas, and there was no evidence for the uptake of synaptic material by activated microglia/macrophages (Figure 3).

### Astrocyte‐destructive lesions outside the hippocampus may cause retrograde axonal degeneration in the hippocampus

**FIGURE 4:**
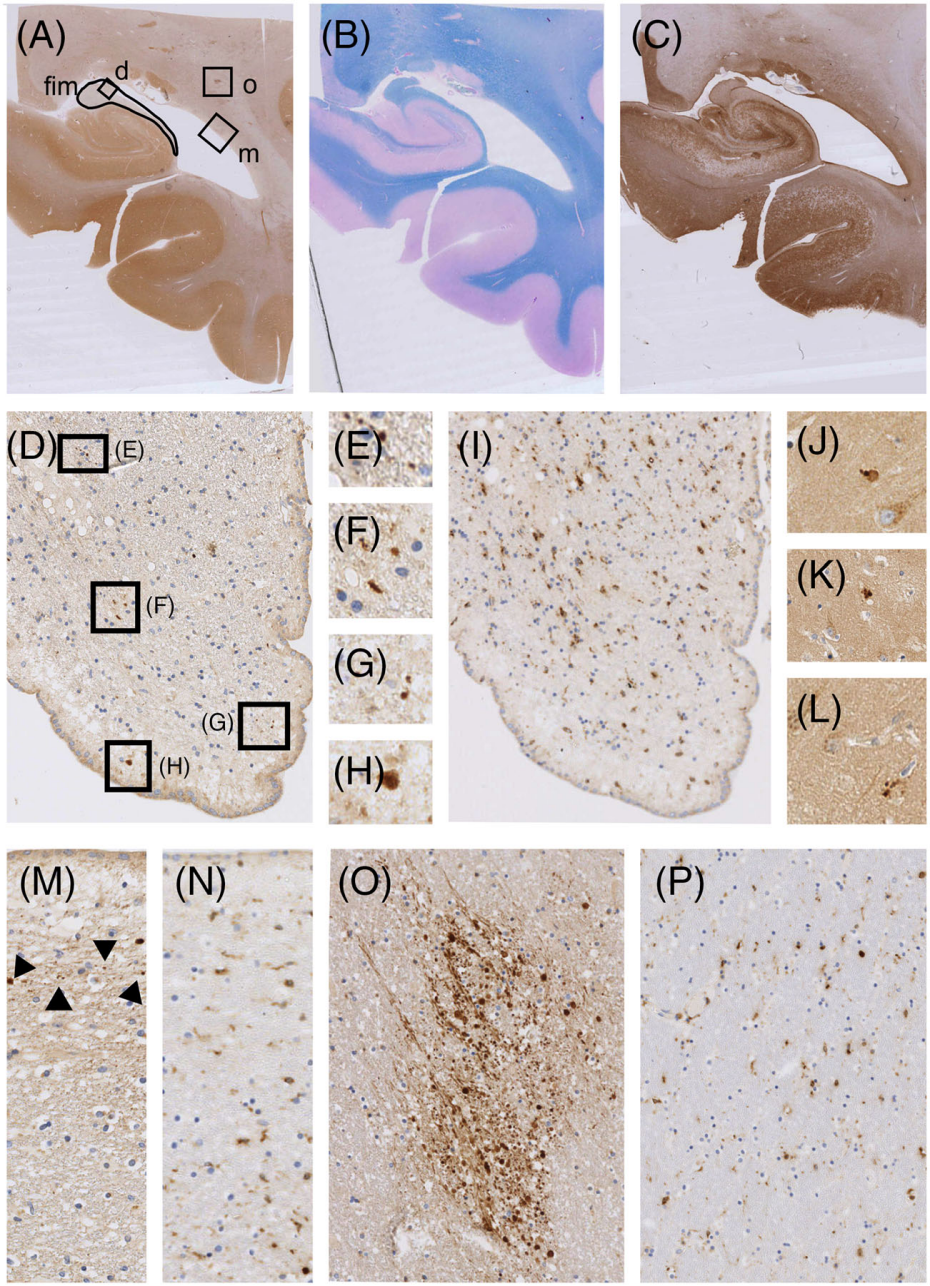
Retrograde axonal degeneration in the deep white matter of the cerebrum and hippocampus in human neuromyelitis optica spectrum disorders. Patient NMO02. Shown here is a tissue block containing the left hippocampus with dentate gyrus and adjacent entorhinal, transentorhinal and temporal cortex. There was no evidence of hypoxic neuronal damage. Adjacent tissue sections were reacted with anti‐APP antibodies (A, D–H, J–L, M, O; brown), subjected to Kluever–Barrera staining (B, myelin sheaths turquoise) or stained with anti‐AQP4 (C, brown) or anti‐CD68 antibodies (I, N, P). Areas with APP^+^ structures (A, D) are boxed and enlarged in (D–H) and (M–P). (F) Note the presence of APP^+^ structures in the fimbria (A, D–L), in the tapetum (A, M [arrowheads]) and in the deep white matter of the cerebrum (A, O). These structures are not associated with microgliosis or microglia nodules (I, N, P) and occur in the absence of demyelination (B) and of aquaporin 4 (AQP4) loss (C). Occasionally, APP^+^ structures were also seen in the Cornu Ammonis 1 region (J–L).

The hippocampus of patient NMO02 displayed normal AQP4 and EAAT2 reactivity throughout the hippocampus, and Kluever–Barrera staining confirmed the presence of seemingly normal myelin sheaths around the axons. CD68^+^ microglial cells were regularly distributed throughout the hippocampus (Figure 4), as also seen in the age‐matched control (data not shown). However, in NMO02, we found APP^+^ spheroids and/or endbulbs in the fimbria hippocampi and, occasionally, APP^+^ structures in the CA1 region (Figure 4). Evidence for neuroaxonal degeneration was also found in the otherwise normal‐appearing tapetum (Figure 4), which connects the hippocampi bilaterally and counts among the long‐range major hippocampal pathways [41]. APP^+^ axonal tracts and spheroids were further noted in the deep white matter of the cerebrum, again in the absence of AQP4 loss or of demyelination (Figure 4). Because this patient had multiple, in part necrotic and demyelinated lesions with loss of AQP4 and loss of GFAP reactivity throughout the entire spinal cord, mostly affecting white matter, and because she also had single tissue‐destructive NMOSD‐typical lesions in the medulla at the level of the lemniscus medialis, in the thalamus lateral to the substantia nigra, in the midbrain at the level of the crus cerebri, in the tegmentum pontis and in the floor of the fourth ventricle (Table 1), the APP^+^ axonal tracts and spheroids in the deep white matter of the cerebrum and in the tapetum point to retrograde axonal degeneration affecting many different axonal tracts.

**FIGURE 5:**
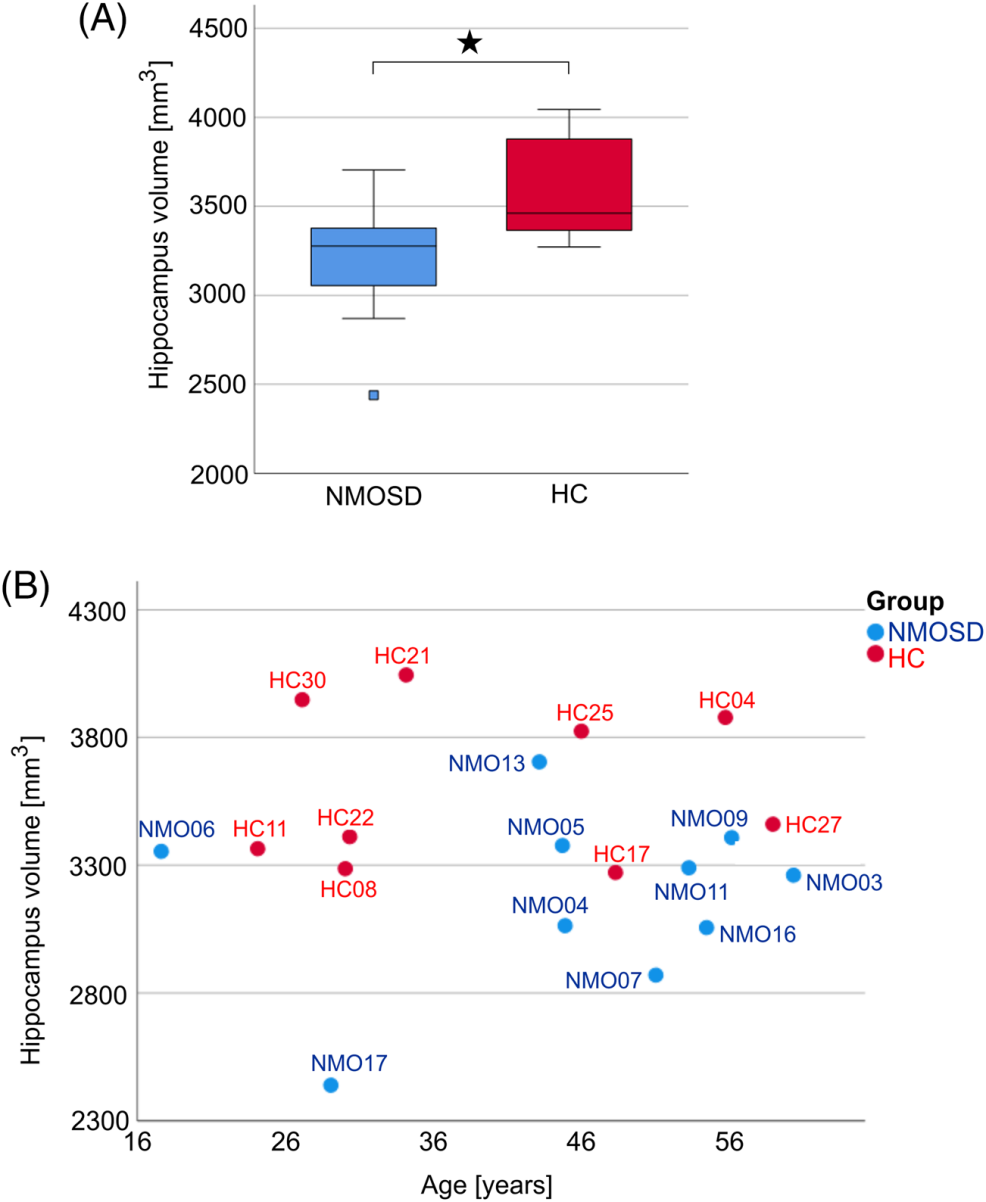
Magnetic resonance imaging of human neuromyelitis optica spectrum disorders (NMOSD). (A) Boxplot of hippocampal volumes (absolute values in mm^3^) in NMOSD patients (blue) and healthy control (HC) subjects (red). Boxes represent the first and third quartiles, the bar inside the box and the bars of the whiskers represent the median, and minimum and maximum values, respectively, with an outlier shown as a single square outside of 1.5 interquartile ranges from the lower end of the box. The significance level of the *t*‐test comparison between means denoted with an asterisk (*) indicates a *p*‐value < 0.05 (exact value: *p* = 0.012). (B) Hippocampal volumes (mm^3^) in relation to age (years) of individual NMOSD patients (blue) and healthy controls (red).

The findings described above suggested that damaged axonal tracts and resulting atrophy could present important pathological substrates for volume loss in the hippocampus. To further substantiate this hypothesis, we compared the hippocampi of NMOSD patients and controls by MRI. This revealed that the NMOSD patient cohort overall had lower hippocampal volumes than the control cohort (Figure 5), in the absence of detectable inflammatory hippocampal lesions at the time of MRI. In the next step, we related the hippocampal volume of individual patients to disease history and lesion characteristics at the time of MRI (Table 1), using a hippocampal volume of 3300 mm^3^ as an arbitrarily selected cut‐off, and a categorisation of hippocampi as having ‘normal volumes’, when they were above, and as having ‘low volumes’ when they were equal or below this threshold. This threshold essentially assigned ‘normal hippocampal volumes’ to healthy controls and used the midline of the graph for easy orientation.

Of the six NMOSD patients with low hippocampal volumes, two had atrophy of optic nerves and chiasm (NMO03 and NMO11), three had atrophy of optic nerves (NMO07, NMO16 and NMO17) and one had spinal cord lesions only, with an Expanded Disability Status Scale (EDSS) of 6.5 after 3 years of disease duration, indicating neuroaxonal damage (NMO04). Three patients with atrophy of optic nerves with or without chiasmatic involvement presented with additional residual clinical deficits, suggesting persisting damage to spinal tracts at the time of MRI (NMO03, NMO11 and NMO17). On the other hand, NMOSD patients with ‘normal volumes’ had NMOSD of extremely short duration (NMO06 and NMO09), area postrema syndrome only (NMO13) or spastic paraparesis after longitudinally extensive transverse myelitis involving the spinal cord segments C2–C5 (NMO05) (Figure 5).

Hence, atrophy of optic nerves and/or damaged spinal tracts were common features among the NMOSD patients with low hippocampal volumes.

## DISCUSSION

We identified different pathological substrates for hippocampal damage in NMOSD and its experimental models.

Damage to the hippocampus can be initiated when hippocampal astrocytes are targeted by pathogenic AQP4‐abs and when these cells are then destroyed by antibody‐dependent and/or complement‐dependent cytotoxicity. We observed an older, chronic NMOSD lesion with AQP4 loss in the hippocampal CA1/2 region of patient NMO01 who was diagnosed with mild cognitive impairment and delusions at a time when her computed tomography (CT) indicated normal brain and hippocampal volumes. We also observed early acute NMOSD‐typical lesions with inflammatory infiltrates, astrocyte destruction and neuroaxonal dysfunction/loss in the hippocampus of the LEW, BN and RNU rat strains used as experimental NMOSD models, which strongly suggests that the genetic background is irrelevant for the formation of hippocampal lesions with AQP4 loss. However, in rats, it seems to play a role in the location of lesions within the dorsal or ventral hippocampus, because BN and RNU rats showed more lesions in the dorsal hippocampus, whereas LEW rats had lesions almost exclusively in the ventral part. Such differences might have consequences for the final outcome of the lesions because dorsal lesions could interfere more with cognitive processes and memory functions, whereas ventral lesions could bear upon emotional processing, affect and the regulation of stress [42]. In principle, however, AQP4‐abs‐induced damage could take root anywhere in the hippocampus, as long as the antibodies gain access to their targets. The size of the hippocampal subfields and the local expression levels of AQP4 were good predictors for lesion formation at specific sites: The largest areas of AQP4 loss were located within the largest hippocampal subfields, that is, the subiculum, DG, CA1 and the CA1/DG border (fissure), whereas lesions with AQP4 loss were essentially absent from the smallest subfields, that is, CA2 and CA4. Within the subfields, most lesions were confined to the CA1 SLM, the DG ML and the dorsal hippocampal fissure. These findings are in line with the laminar specificity of hippocampal AQP4 immunoreactivity, which is highest at these sites [43]. They are also in line with a recent MRI study on human NMOSD hippocampal subfields detecting loss of subfield volumes in the subiculum, DG and CA1 [18].

NMOSD‐typical, astrocyte‐destructive lesions in the hippocampus could have many different consequences:

1. Astrocytes are important for water homeostasis in the CNS, and their destruction may trigger an iono‐osmotic imbalance initiating axon pathology [44]. Moreover, astrocytes normally phagocytose and eliminate redundant excitatory hippocampal synapses [45], which leads within a few weeks to a full erasure of connectivity patterns in the adult CA1 [46]. Therefore, astrocyte loss at this site could interfere with the synaptic turnover needed for circuit homeostasis. Loss of astrocytes and/or loss of astrocytic AQP4 expression also leads to a loss of astrocytic EAAT2 reactivity [47], as also demonstrated in patient NMO01. Loss of this transporter could cause the overactivation of glutamate receptors in postsynaptic neurons [48], which may culminate in excitotoxic tissue injury. This phenomenon has already been described in genetically modified mice, where a deficiency of EAAT2 resulted in selective neuronal degeneration in the hippocampal CA1 pyramidal layer [49]. Loss of EAAT2 in CA1 can also interfere with synaptic plasticity [50, 51]: In the intact hippocampus, high‐frequency neuronal activity normally induces a brief, local enhancement of glutamatergic excitation via inhibition of astrocytic glutamate transport [48], and this inhibition is slowed by a loss of EAAT2 expressing astrocytes [52]. Abnormal use‐dependent synaptic plasticity is considered to be the main physiological correlate of memory deficits [53].
2. Microglial activation and an open blood–brain barrier allowing entry of serum complement components in early lesions could jointly impede hippocampal circuits because complement‐dependent phagocytosis of synapses by microglia plays an important role in hippocampal engram dissociation and memory forgetting [54].
3. The large quantities of neutrophils, eosinophils, activated microglia, macrophages and T cells [40] seen in early NMOSD lesions may generate a pro‐inflammatory milieu culminating in the activation of interleukin 1 and 6 (IL‐1 and IL‐6) signalling cascades [30, 55]. Exposure of hippocampal slices to IL‐1β reportedly causes alterations in hippocampal synaptic plasticity rules, resulting in a favouring of the induction of long‐term potentiation over long‐term depression [53]. Elevated IL‐1β levels in the CNS of animals with neuroinflammation cause the same abnormal use‐dependent synaptic plasticity, along with a selective loss of GABAergic interneurons and a reduction of gamma‐frequency oscillations in the hippocampal CA1 region, which may impair memory storage and retrieval [53, 56]. IL‐6 elevation in the hippocampus may stimulate the release of excitatory neurotransmitters [57, 58], alter the balance of excitatory and inhibitory processes and, by doing so, may further contribute to cognitive impairment [58].
4. External inputs to the hippocampus coming from the cortex, thalamus and corpus mamillare might be reduced by lesions at the CA1 SLM and the DG ML [42].

The changes in circuits and plasticity caused by astrocyte damage, neuroaxonal dysfunction/damage and pro‐inflammatory cytokines are acting in early, active lesions, and the extent to which these changes persist during remission remains unclear. Once inflammation and the production of pro‐inflammatory cytokines subside and once lesion repair is initiated, destroyed astrocytes could be replaced by invading astrocytes from the lesion border. However, neuronal loss from most parts of the hippocampus would be permanent, with a potential exception of the human DG region where adult neurogenesis may occur [59, 60].

Experimental animals with ongoing/established subependymal hippocampal lesions also displayed scattered activated microglial cells, predominantly throughout the DG. One likely reason for this finding is the presence of numerous granule cell dendrites in close vicinity to damaged astrocytes in the subependymal lesions. Because astrocyte loss equates to the loss of EAAT2, elevated concentrations of glutamate could result in the excessive activation of glutamate receptors on granule cells. This might first induce increased granule cell activation followed by the spread of excitation over mossy fibre‐CA3 pyramidal neuron (detonator) synapses enabling aberrant CA3 and DG activity [61, 62] and might finally result in excitotoxic death of granule cells. Because microglial responses to neuronal stimulation only occur at early postnatal ages and not in the adult [63], it is tempting to speculate that the scattered ED1^+^ microglial cells found throughout the DG have been activated by excitotoxic damage to granule cells. Remote microglial activation has been described before in models of focal brain injury [64, 65, 66] and after stroke in rats [67] and humans [68].

Our study revealed that the hippocampus may also become compromised when astrocyte‐destructive lesions have formed at remote places like the optic nerves or the spinal cord. The irreparable local damage to axons may then spread to axonal tracts and neuronal networks by subsequent retrograde neuronal degeneration and may then culminate in volume loss of the hippocampus. The first indications for such a scenario come from the pathological analysis of the hippocampal area of patient NMO02. She did not have typical NMOSD lesions in the hippocampus but had lesions with AQP4 loss at other sites of the brain and numerous, in part necrotic lesions throughout the entire spinal cord. She also showed degenerating axonal tracts at sites with apparently normal myelin and normal AQP4 reactivity, that is, in the deep white matter of the cerebrum and in the tapetum, which borders the splenium of the corpus callosum, connects the hippocampi bilaterally and counts among the long‐range major hippocampal pathways [41]. NMO02 also had APP^+^ spheroids and/or endbulbs in the fimbria hippocampi. Cumulatively, these data indicated that retrograde axonal degeneration in NMOSD may affect many different axonal tracts and could affect both input and output hippocampal connections. This finding was further corroborated by the MRI analysis of patients NMO03–NMO17, which clearly revealed that atrophy of optic nerves and/or damaged spinal tracts were common features among NMOSD patients with low hippocampal volumes, but not among NMOSD patients with normal hippocampal volumes. The loss of hippocampal volume in patients with optic nerve atrophy cannot be explained by an absence of visual input, because blind individuals have an overall volume increase of the hippocampus when compared to normal sighted matched controls, most likely due to adaptive responses to sensory deprivation [69, 70]. Hence, it is more likely that the hippocampal volume loss in NMOSD patients with atrophy of optic nerves and/or damaged spinal tracts is a consequence of damage to the connections of the hippocampus from and to other regions of the brain. This conclusion is in line with reports relating cognitive impairment of NMOSD patients to brain white matter atrophy [3], to a decrease in grey matter volume [8, 11, 13, 71], or to neuronal loss in the cortex [7], and is in line with descriptions that cognitive impairment occurred both in the presence [1, 4, 72] and in the absence of brain lesions [73], seemingly independent of other symptoms, disease exacerbation and the extent of physical disability [74, 75]. It is also compatible with a report about the progression of brain atrophy in NMOSD [76]. Our conclusion is further substantiated by reports about changes in structural networks [77, 78], functional connectivity alterations [79, 80], network maladaptations [81] and network disruptions [82] in the brains of NMOSD patients.

Retrograde degeneration of axonal tracts causing first changes or disruptions of networks and functional connectivity and culminating later in hippocampal volume loss could also explain why some groups could not find any relation between cognitive impairment and white/grey matter size in NMOSD [9, 10, 12, 73], or why some groups find a loss of hippocampal volume [16, 17, 18, 19] in NMOSD patients, whereas others describe cognitive impairment independent of hippocampal size [13, 20]. It is tempting to speculate that the differences in hippocampal size changes between these NMOSD patient cohorts might be explainable by variable degrees of retrograde axonal degeneration, possibly caused by differences in usage, timing, duration and effectiveness of therapies during lesion attack.

Further and more refined MRI studies would be needed to solve this issue.

In the end, it might be interesting to note that NMOSD patients with large spinal cord lesions resemble patients with spinal cord injury (SCI) much more than previously appreciated. Also, many SCI patients suffer from cognitive impairment and depression [83] and show a progressive reduction of grey matter volume in regions that are not directly connected to the injury site but that play a crucial role in attention and processing of emotional information [84, 85, 86]. Moreover, SCI in experimental animals also resulted in significant neuronal loss in the cortex, hippocampus and thalamus when the animals were evaluated in the chronic phase, but not when they were studied at early time points after SCI [85, 87]. Such experiments also revealed reduced neuronal survival and higher numbers of activated microglial cells in the cortex and hippocampus of spinal cord‐injured animals, but only when the animals experienced moderate or severe rather than mild trauma [88].

### Limitations of the present study

Our study has some limitations. The NMOSD patients evaluated by pathology were different from those who underwent MRI. Both of these cohorts were evaluated retrospectively, and with one exception (NMO01), information about the cognitive function or cognitive tests of these patients is not available. Hence, future prospective studies with long‐term follow‐up and standardised cognitive analysis are warranted. Moreover, only a few human NMOSD hippocampi were available for pathological studies, owing to the preferential routine archiving of medulla/spinal cord samples of NMOSD patients. The animals from the ‘antibody‐only’ and the ‘antibody plus T cell’ rat models were studied in the early/acute phase of their disease. In both, the late phases of the disease were unavailable. The reasons for this are twofold. First, the animal models were induced with NMOSD‐patient‐derived NMO‐IgGs as a source of pathogenic AQP4‐specific antibodies or with a murine monoclonal AQP4‐specific antibody. Both sources are xenogeneic for rats and will induce immune responses against the ‘foreign’ AQP4‐specific antibodies in a short time. Rats producing ‘their own’ pathogenic, AQP4‐specific antibodies over a longer period of time are not available. Second, in the ‘antibody‐only’ model, very high antibody titres were reached [24], and the pathological changes induced in these animals were not compatible with long‐term survival to reach a late stage of the disease. We also did not perform cognitive tests on these sick animals. Although plasmapheresis to reduce/remove antibodies from the circulation, as done in NMOSD patients, would also be doable in rats, it would substantially increase the stress level of the animals with a high risk to provoke stress‐induced changes to the hippocampus [42] different from NMOSD‐induced changes.

We identified different pathological scenarios for hippocampal damage in NMOSD and its experimental models. In the first case, the hippocampus was compromised by the initiation of astrocyte injury in this brain region and subsequent local effects of microglial activation and neuronal damage. In the second case, loss of hippocampal volume was seen by MRI in patients with large tissue‐destructive lesions in the optic nerves or the spinal cord, and the pathological work‐up of tissue derived from a patient with such lesions revealed subsequent retrograde neuronal degeneration affecting different axonal tracts and neuronal networks. It remains to be seen whether remote lesions and associated retrograde neuronal degeneration on their own are sufficient to cause extensive volume loss of the hippocampus, or whether they act in concert with small astrocyte‐destructive, microglia‐activating lesions in the hippocampus that escape detection by MRI, either due to their small size or due to the chosen time window for examination.

## AUTHOR CONTRIBUTIONS

MZ performed the majority of the histological studies of rat hippocampi; DH and SH established and initially characterised the experimental NMOSD models; MP made retrospective MRI analysis of human patients and controls; MN and MP made the statistical analyses for rat hippocampi (MN) and MRI (MP); YT and TM pathologically characterised human NMOSD hippocampi; RH identified and histologically characterised brains and spinal cords of one further NMOSD patient included in the hippocampal analysis; PR and GK diagnosed and characterised patients of the MRI study and critically helped in the project design of the human study; JB and BL gave important input on hippocampal analyses; MB conceived the project; MB, GK and PR supervised the project; and MZ, MN, MP and MB wrote the manuscript. All authors reviewed the manuscript.

## CONFLICT OF INTEREST

MZ, MN, DH, JB, BL, GK, YT and MB declare no conflict of interest. MP has participated in meetings sponsored by and received speaker honoraria or travel funding from Amicus, Merck, Novartis and Sanofi‐Genzyme. PR received honoraria for lectures or consultancy from Amicus, Alexion, Amirall, Biogen, Merck, Novartis, Roche, Sanofi, Sandoz and Teva. He served on advisory boards for Amicus, Alexion, Merck, Roche, Sanofi and Sandoz and received research grants from Amicus, Biogen, Merck and Roche. RH reports speaker's honoraria from Novartis and Biogen. The Medical University of Vienna (Austria; employer of RH) receives payment for antibody assays and antibody validation experiments organised by Euroimmun (Lübeck, Germany). TM received speaker honoraria from Tanabe Mitsubishi Pharma, Novartis Pharma, Alexion Pharma and Biogen Idec Japan and received research support from Cosmic Corporation and Medical and Biological Laboratories (MBL).

## ETHICS STATEMENT

In keeping with the local/national regulations, pathological studies of human hippocampi were approved by the Ethics Committees of the Medical University of Vienna (EK‐No. 1636/2019, which allows usage of remainders from diagnostic material for research, and EK‐No. 1133/2022, which allows the retrospective use of patient data for research) and of the Tohoku University Graduate School of Medicine (No. 2011‐194). In all these cases, there was no need for additional informed consent for research by the patients or guardians. MRI studies on NMOSD patients and healthy controls were again retrospective studies without the need for informed consent, were approved by the Ethics Committee of the Medical University of Vienna (EK 1008/2022; EK 1487/2020) and were conducted in accordance with the 1964 Declaration of Helsinki. All animal studies were approved by the Ethics Commission of the Medical University Vienna and performed with the licence of the Austrian Ministry for Science and Research (GZ: BMBWF‐66.009/0136‐WF/V/3b/2016, BMBWF‐66.009/0107‐V/3b/2018 and BMBWF‐66.009/0221‐V/3b/2018).

## Supporting information


**Table S1:** **Detailed MRI information.**
**Figure S1:** Enlarged view of hippocampal lesions of patient NMO01 shown in figure 1 d (EAAT2 staining) and figure 1 k (CD68 staining) of the core manuscript. The original pictures were rather bright. We therefore made non‐linear gamma adjustments to show the absence of small lobulated blue nuclei indicating neutrophils in the EAAT2‐specific antibody reacted lesions (a), and to show the absence of CD68+ activated microglia/macrophages in the hippocampal parenchyma (b; black arrow heads point to meningeal CD68+ cells, and the open arrow head points to a staining artefact).
**Figure S2:** Distribution of lesions with AQP4 loss in the CNS of Lewis (orange, n = 5), RNU (red, n = 5), and BN rats (blue, n = 5). Shown here are brain and spinal cord [cervical (C1–7), thoracal (T1–10) and lumbar/sacral (L1‐S4)] schemes as well as an outline of optic nerve, chiasm, and optic tract. The animals were analyzed 120 hours after daily intraperitoneal injections of AQP4‐abs, and the location of an established lesion with AQP4 loss was projected in the corresponding color into the schemes provided by Paxinos and Watson [33] as guide lines. Please note that the lesion distribution of Lewis and RNU rats has already been published before [24].
**Figure S3: Lesioned hippocampal subfields.** a) Within‐rat‐group differences of AQP4 loss in different hippocampal subfields along the dorso‐ventral hippocampal axis. For each strain, the area with AQP4 loss was determined in the different subfields (subiculum (SUB), Cornu Ammonis (CA) areas CA1‐CA4, dentate gyrus (DG), mixed CA1/DG subfields, and fimbria (FI)) of 5 rats, and expressed as percentage of the corresponding total dorsal or ventral hippocampal area. The data were analyzed with the Related‐Samples Friedman's Two‐way Analysis of Variance by Ranks. The resulting test values (0.006, 0.012 and 0.001 for the dorsal hippocampus of Brown Norway rats, ventral and dorsal hippocampus of Rowett Nude rats, respectively) indicate statistically significant differences in the distribution of areas with AQP4 loss between two or more hippocampal subfields. b: Global differences of AQP4 loss in different hippocampal subfields along the dorso‐ventral hippocampal axis. For each rat (total n = 15, 5 each per Lewis, Brown Norway and Rowett Nude strain), the area with AQP4 loss was determined in the different subfields (subiculum (SUB), Cornu Ammonis (CA) areas CA1‐CA4, dentate gyrus (DG), mixed CA1/DG subfields, and fimbria (FI)) and expressed as percentage of the corresponding total dorsal or ventral hippocampal area. The data were analyzed with the Independent‐Sample‐Kruskal‐Wallis Test (Omnibus Test). The resulting test values (0.001 for the dorsal, and 0.009 for the ventral hippocampus) indicate that there are statistically significant differences in the distribution of lesioned areas between two or more hippocampal subfields. Please note that for this type of test, data from all rat strains were pooled and therefore subject to intergroup variance.
**Figure S4: Neutrophils and lesion stage/size in coronal brain section at the level of the hippocampus.** All pictures shown here derive from a single BN rat intraperitoneally injected for 5 consecutive days with AQP4‐abs. (a) Tissue section stained with antibodies against AQP4 (brown), and counterstained with hematoxylin to show nuclei (blue). Please note the presence of 2 different perivascular lesions with AQP4 loss, at comparable locations and with comparable sizes. These lesions are enlarged in b) and c). (b) early active lesion, with AQP4 loss and numerous neutrophils (evidenced by their lobulated nuclei) dispersed throughout the lesion. (c) late lesion which still displays AQP4 loss but lacks neutrophils. On the upper left outside this lesion, neutrophils are seen in an area with ongoing loss of AQP4 reactivity. This picture is reminiscent of our previous findings that established lesion in the brain precipitate lesion formation in the vicinity [24 of the core manuscript]. (d‐f) Subpial lesions with AQP4 loss and high (d), intermediate (e), and very low (f) numbers of neutrophils in the meninges above.
**Figure S5: Scattered activated microglial cells in the hippocampus of BN rats.** Coronal sections at the level of the hippocampus derived from BN rats were reacted with the antibody ED1 to identify activated microglia/macrophages (brown) and counterstained with hematoxylin to show nuclei (blue). The animals had been injected daily for 5 consecutive days with AQP4‐abs and were analyzed 24 hours after the last injection. In the presence of remote hippocampal subependymal lesions with AQP4 loss, scattered ED1^+^ microglial cells/macrophages are seen in the dentate gyrus (a). These cells are undetectable in the absence of such lesions.

## Data Availability

The data that support the findings of this study are available from the corresponding author upon reasonable request.
