# Supplementary material for: Paths to hippocampal damage in neuromyelitis optica spectrum disorders
Source: Neuropathol Appl Neurobiol. 2023 Mar 6;49(2):e12893. doi: 10.1111/nan.12893 (PMC10947283; doi:10.1111/nan.12893)
Supplement: Supplementary file 1 — Table S1: Detailed MRI information. Figure S1: Enlarged view of hippocampal lesions of patient NMO01 shown in figure 1 d (EAAT2 staining) and figure 1 k (CD68 staining) of the core manuscript. The original pictures were rather bright. We therefore made non‐linear gamma adjustments to show the absence of small lobulated blue nuclei indicating neutrophils in the EAAT2‐specific antibody reacted lesions (a), and to show the absence of CD68+ activated microglia/macrophages in the hippocampal parenchyma (b; black arrow heads point to meningeal CD68+ cells, and the open arrow head points to a staining artefact). Figure S2: Distribution of lesions with AQP4 loss in the CNS of Lewis (orange, n = 5), RNU (red, n = 5), and BN rats (blue, n = 5). Shown here are brain and spinal cord [cervical (C1–7), thoracal (T1–10) and lumbar/sacral (L1‐S4)] schemes as well as an outline of optic nerve, chiasm, and optic tract. The animals were analyzed 120 hours after daily intraperitoneal injections of AQP4‐abs, and the location of an established lesion with AQP4 loss was projected in the corresponding color into the schemes provided by Paxinos and Watson [33] as guide lines. Please note that the lesion distribution of Lewis and RNU rats has already been published before [24]. Figure S3: Lesioned hippocampal subfields. a) Within‐rat‐group differences of AQP4 loss in different hippocampal subfields along the dorso‐ventral hippocampal axis. For each strain, the area with AQP4 loss was determined in the different subfields (subiculum (SUB), Cornu Ammonis (CA) areas CA1‐CA4, dentate gyrus (DG), mixed CA1/DG subfields, and fimbria (FI)) of 5 rats, and expressed as percentage of the corresponding total dorsal or ventral hippocampal area. The data were analyzed with the Related‐Samples Friedman's Two‐way Analysis of Variance by Ranks. The resulting test values (0.006, 0.012 and 0.001 for the dorsal hippocampus of Brown Norway rats, ventral and dorsal hippocampus of Rowett Nude rats, respectively) [file NAN-49-0-s001.docx]

**Paths to hippocampal damage in NMOSD**

Mona Zakani^1^, Magdalini Nigritinou^1^, Markus Ponleitner^2^, Yoshiki Takai^3^, Daniel Hofmann^1^, Sophie Hillebrand^1^, Romana Höftberger^4^, Jan Bauer^1^, Balint Lasztoczi^5^, Tatsuro Misu^3^, Gregor Kasprian^6^, Paulus Rommer^2^, and Monika Bradl^1*^

**Supplementary table 1: Detailed MRI information**

|  | NMOSD | Healthy Controls |
| --- | --- | --- |
| 3 Tesla field strength | 10/10 (100%)³ | 9/9 (100%)³ |
| Voxel spacing (mm) | 0.49 (0.16; 0.23 – 0.72)² | 0.23 (0)^1^ |
| Slice thickness (mm) | 2.47 (0.47; 2.0 – 3.0)² | 2.00 (0)^1^ |
| Echo Time (ms) | 103.2 (14.6; 84 – 119)² | 96 (0)^1^ |
| Repetition Time (ms) | 4862.6 (1042.6; 3060 – 6300)² | 4500.0 (0)^1^ |

^1^ Mean (standard deviation)
² Mean (standard deviation; range min – max)
³ Number (percentage)


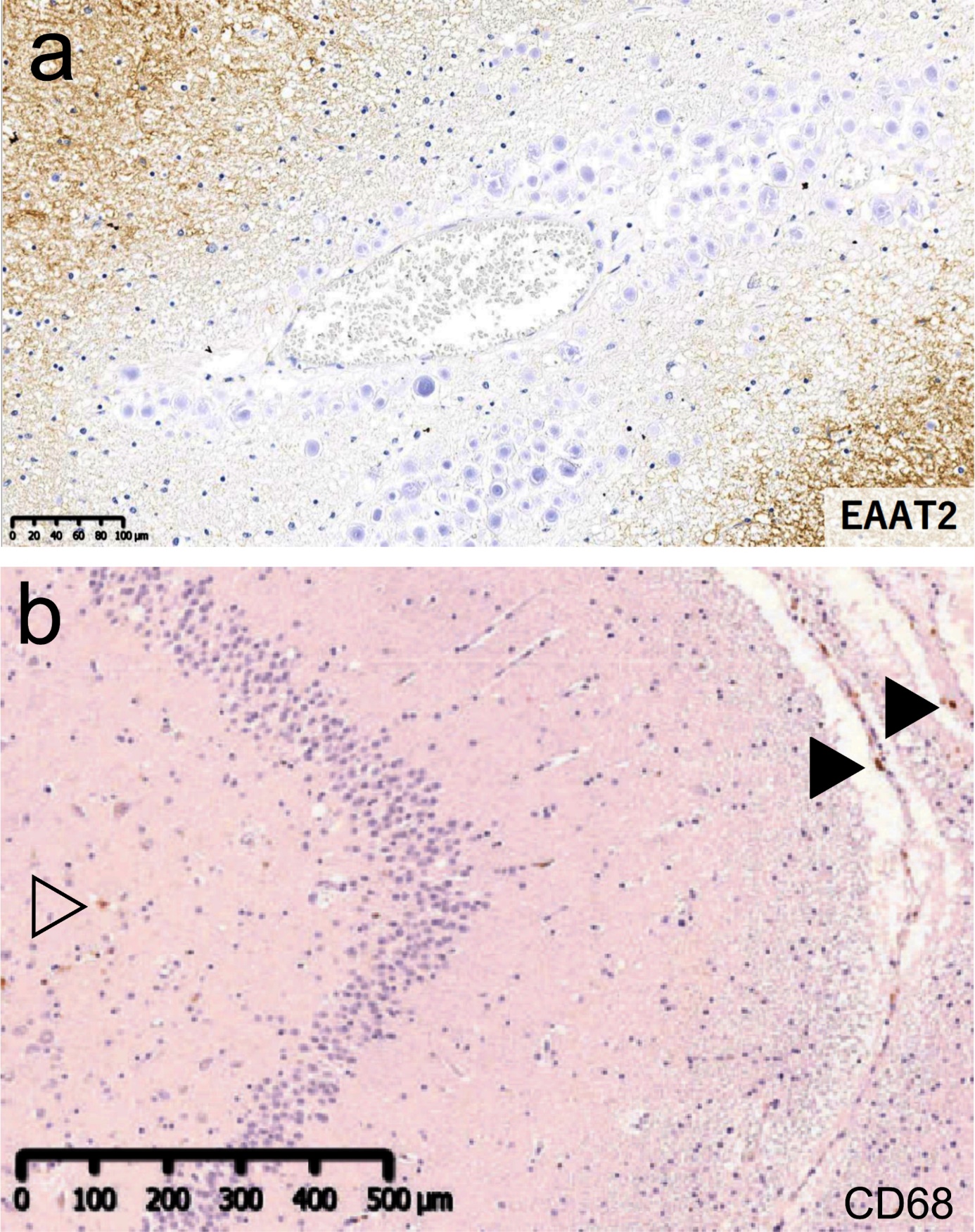


**Supplementary fig. 1:** Enlarged view of hippocampal lesions of patient NMO01 shown in fig. 1 d (EAAT2 staining) and fig. 1 k (CD68 staining) of the core manuscript. The original pictures were rather bright. We therefore made non-linear gamma adjustments to show the absence of small lobulated blue nuclei indicating neutrophils in the EAAT2-specific antibody reacted lesions (a), and to show the absence of CD68+ activated microglia/macrophages in the hippocampal parenchyma (b; black arrow heads point to meningeal CD68+ cells, and the open arrow head points to a staining artefact).

**
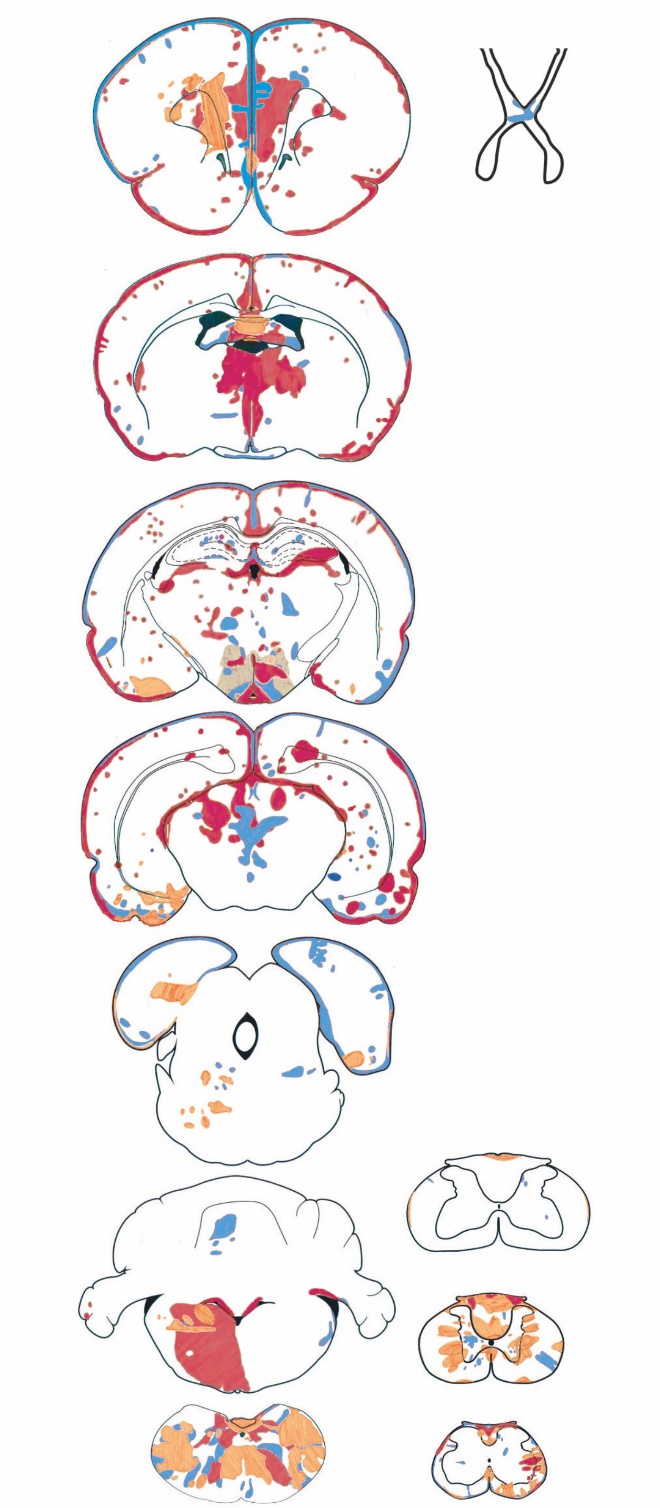
**

**Supplementary fig. 2:** Distribution of lesions with AQP4 loss in the CNS of Lewis (orange, n=5), RNU (red, n=5), and BN rats (blue, n=5). Shown here are brain and spinal cord [cervical (C1-7), thoracal (T1-10) and lumbar/sacral (L1-S4)] schemes as well as an outline of optic nerve, chiasm, and optic tract. The animals were analyzed 120 hours after daily intraperitoneal injections of AQP4-abs, and the location of an established lesion with AQP4 loss was projected in the corresponding color into the schemes provided by Paxinos and Watson [33] as guide lines. Please note that the lesion distribution of Lewis and RNU rats has already been published before [24].


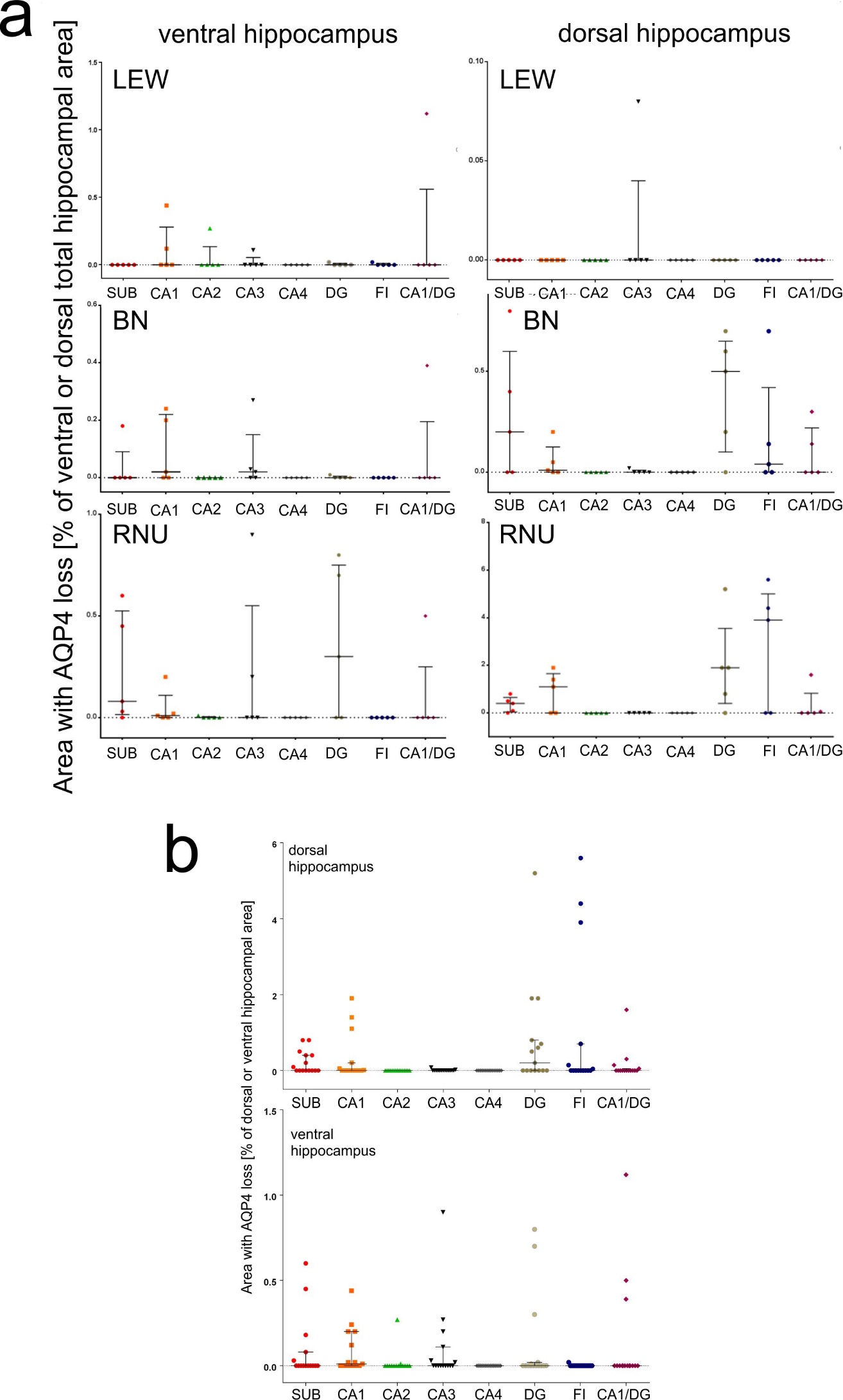


**Supplementary fig. 3: Lesioned hippocampal subfields**

a) Within-rat-group differences of AQP4 loss in different hippocampal subfields along the dorso-ventral hippocampal axis. For each strain, the area with AQP4 loss was determined in the different subfields (subiculum (SUB), Cornu Ammonis (CA) areas CA1-CA4, dentate gyrus (DG), mixed CA1/DG subfields, and fimbria (FI)) of 5 rats, and expressed as percentage of the corresponding total dorsal or ventral hippocampal area. The data were analyzed with the Related-Samples Friedman´s Two-way Analysis of Variance by Ranks. The resulting test values (0.006, 0.012 and 0.001 for the dorsal hippocampus of Brown Norway rats, ventral and dorsal hippocampus of Rowett Nude rats, respectively) indicate statistically significant differences in the distribution of areas with AQP4 loss between two or more hippocampal subfields.

b: Global differences of AQP4 loss in different hippocampal subfields along the dorso-ventral hippocampal axis. For each rat (total n=15, 5 each per Lewis, Brown Norway and Rowett Nude strain), the area with AQP4 loss was determined in the different subfields (subiculum (SUB), Cornu Ammonis (CA) areas CA1-CA4, dentate gyrus (DG), mixed CA1/DG subfields, and fimbria (FI)) and expressed as percentage of the corresponding total dorsal or ventral hippocampal area. The data were analyzed with the Independent-Sample-Kruskal-Wallis Test (Omnibus Test). The resulting test values (0.001 for the dorsal, and 0.009 for the ventral hippocampus) indicate that there are statistically significant differences in the distribution of lesioned areas between two or more hippocampal subfields.

Please note that for this type of test, data from all rat strains were pooled and therefore subject to intergroup variance.


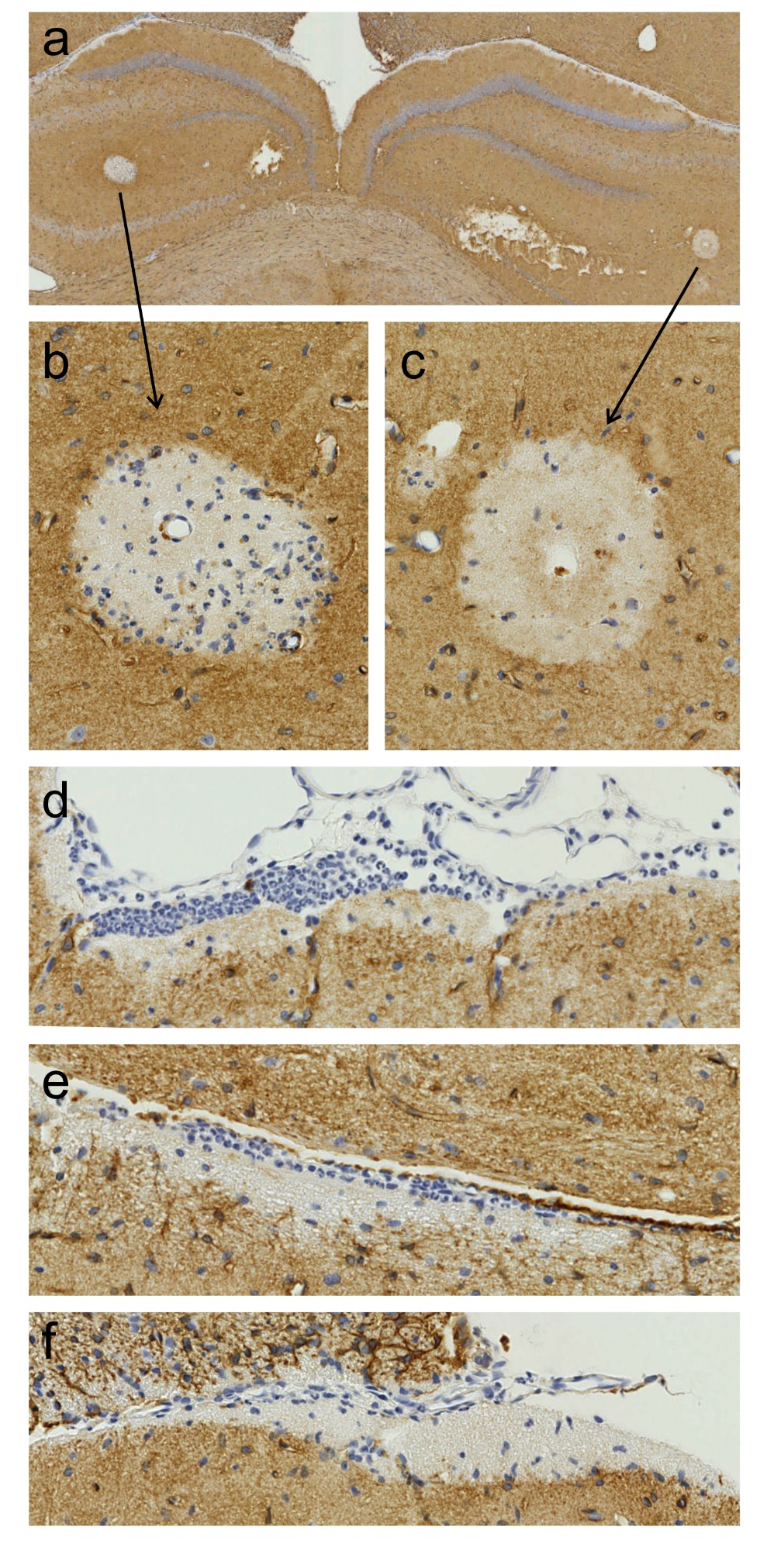


**Supplementary fig. 4: Neutrophils and lesion stage/size in coronal brain section at the level of the hippocampus**

All pictures shown here derive from a single BN rat intraperitoneally injected for 5 consecutive days with AQP4-abs.

(a) Tissue section stained with antibodies against AQP4 (brown), and counterstained with hematoxylin to show nuclei (blue). Please note the presence of 2 different perivascular lesions with AQP4 loss, at comparable locations and with comparable sizes. These lesions are enlarged in b) and c).

(b) early active lesion, with AQP4 loss and numerous neutrophils (evidenced by their lobulated nuclei) dispersed throughout the lesion.

(c) late lesion which still displays AQP4 loss but lacks neutrophils. On the upper left outside this lesion, neutrophils are seen in an area with ongoing loss of AQP4 reactivity. This picture is reminiscent of our previous findings that established lesion in the brain precipitate lesion formation in the vicinity [24 of the core manuscript].

(d-f) Subpial lesions with AQP4 loss and high (d), intermediate (e), and very low (f) numbers of neutrophils in the meninges above.

Cumulatively, these data show that the numbers of neutrophils associated with loss of AQP4 reactivity profoundly differ in lesions of single animals, making it impossible to correlate lesion stages with neutrophil numbers and lesion extension.


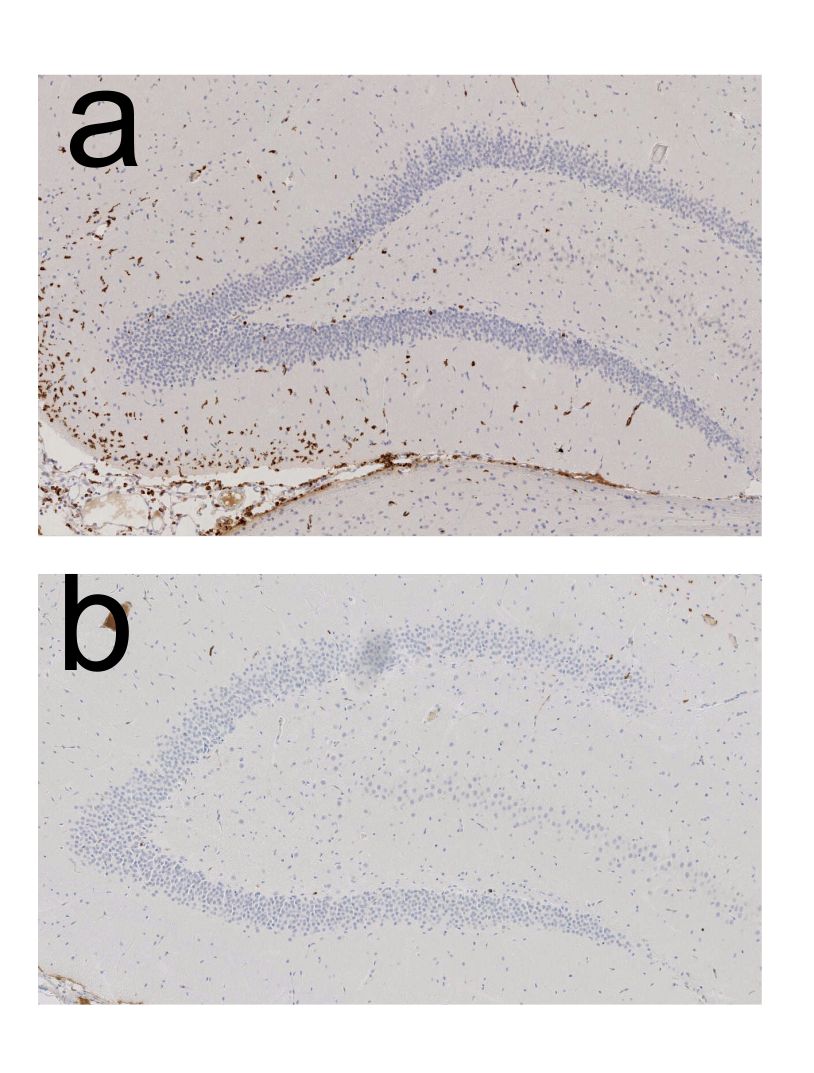


**Supplementary fig. 5: Scattered activated microglial cells in the hippocampus of BN rats**

Coronal sections at the level of the hippocampus derived from BN rats were reacted with the antibody ED1 to identify activated microglia/macrophages (brown) and counterstained with hematoxylin to show nuclei (blue). The animals had been injected daily for 5 consecutive days with AQP4-abs and were analyzed 24 hours after the last injection. In the presence of remote hippocampal subependymal lesions with AQP4 loss, scattered ED1^+^ microglial cells/macrophages are seen in the dentate gyrus (a). These cells are undetectable in the absence of such lesions.
